# Isolation, Bromination, and Antimicrobial Activity of 3-Ethyl-4-hydroxy-6-methyl-*2H*-pyran-2-one from Sea Cucumber-Associated Fungus *Trichoderma koningii* KMM 4751

**DOI:** 10.3390/antibiotics15060554

**Published:** 2026-05-30

**Authors:** Sofya S. Starnovskaya, Dmitry N. Pelageev, Ekaterina A. Chingizova, Natalya N. Kirichuk, Yulia V. Khudyakova, Konstantin A. Drozdov, Ekaterina A. Yurchenko, Anton N. Yurchenko

**Affiliations:** G.B. Elyakov Pacific Institute of Bioorganic Chemistry FEB RAS, 690022 Vladivostok, Russia; pelageev@piboc.dvo.ru (D.N.P.); chingizova_ea@piboc.dvo.ru (E.A.C.); kirichuk_nn@piboc.dvo.ru (N.N.K.); 161070@rambler.ru (Y.V.K.); drozdovka@piboc.dvo.ru (K.A.D.); eyurch@piboc.dvo.ru (E.A.Y.); yurchenkoan@piboc.dvo.ru (A.N.Y.)

**Keywords:** marine fungi, *Trichoderma koningii*, secondary metabolites, biological activity

## Abstract

**Objectives**: This study aimed to isolate secondary metabolites from sea cucumber-associated fungus *Trichoderma koningii* KMM 4751, obtain their bromine derivatives, and investigate their antimicrobial and cytotoxic activities. **Results**: 3-Ethyl-4-hydroxy-6-methyl-*2H*-pyran-2-one (EHMP) was isolated from fungal extract. It was brominated, and a previously unreported 6-(bromomethyl)-3-ethyl-4-hydroxy-*2H*-pyran-2-one (Br-EHMP) was obtained. EHMP inhibited the formation of *Candida albicans* biofilms with an IC_50_ of 49.3 µM, but Br-EHMP was less active. Simultaneously, bromination of EHMP significantly enhanced the inhibitory effect of Br-EHMP on *Staphylococcus aureus* growth and biofilm formation without increasing cytotoxicity to H9c2 cells. Br-EHMP at 10 μM can inhibit sortase A activity by near 30% in a cell-free assay. In silico molecular docking predicted the interaction of Br-EHMP with Cys184 in the sortase A active site. **Conclusions**: Br-EHMP emerges as a promising antibiofilm agent, and its mechanism involves sortase A inhibition.

## 1. Introduction

Marine fungi are a promising source of novel compounds, primarily antimicrobials, for drug discovery. Between 2012 and 2023, 223 antimicrobial compounds were identified [1]. However, drug discovery involves not only the isolation of compounds but also their optimization to obtain more active and drug-like structures. For example, the natural antibiotic penicillin G was modified to temocillin by introducing a methoxy group at the 6a-position in the nucleus [2]. Another method of semisynthetic modification of natural compounds is bromination, which has been reported as a possible tool for the optimization of compounds to enhance their lipophilicity or increase the possibility of intermolecular bond formation (attractive interactions) between the electrophilic region of the molecule containing bromine atoms and various nucleophilic active sites of biomolecules [3]. Brominated compounds may exhibit more pronounced antimicrobial and antibiotic properties than their nonbrominated counterparts [4]. 2,7-dibromophenanthrenequinone and 3,6-dibromophenanthrenequinone disrupted *Staphylococcus aureus* biofilms more effectively than their nonbrominated counterparts [5]. Lead molecule optimization methods may be empirical or calculated using local QSAR models, chemical transformation rules (as implemented in the OptADMET web tool [6]), and emerging AI-based approaches (e.g., SynMap module in the Syntelly platform [7]).

*Trichoderma koningii* is widely recognized as an effective biocontrol agent against various phytopathogenic fungi. Its inhibitory activity is mediated by diverse mechanisms, including mycoparasitism, antibiosis, and enzyme production [8]. Specific secondary metabolites, including 6-pentyl-α-pyranone, benzopyranone antibiotics, and peptaibols, exhibit broad-spectrum antimicrobial activity comparable to that of commercial fungicides [9]. However, marine-derived isolates of *T. koningii* remain an under-researched source of chemical diversity. Unlike their terrestrial counterparts, these strains have adapted to distinct environmental selective pressures, such as high salinity, pressure, and nutrient competition, which activate adaptive biosynthetic pathways [10]. Despite this potential, only a few polyketides have been described from marine strains of *T. koningii*, including trichodermaketone A, which inhibits the growth of *Candida albicans* in synergy with ketoconazole [11], antistaphylococcal hypofuran A [12], and several tyrosol derivatives, such as hypocrol A, which inhibit the growth of *Staphylococcus aureus* and *Escherichia coli* [13].

We obtained the *T. koningii* strain KMM 4751 from the intestines of the sea cucumber *Apostichopus japonicus*, which was collected in Peter the Great Bay in the Sea of Japan.

This study aimed to isolate secondary metabolites from *Trichoderma koningii* KMM 4751, obtain their bromine derivatives, and investigate their antimicrobial and cytotoxic activities.

## 2. Results

### 2.1. Culture Condition Study

Based on the comparison of metabolite production in various analytical cultivations (Table S2, Figure S1), *T. koningii* KMM 4751 was subsequently scaled up and cultured on solid rice medium for 21 days to isolate individual compounds.

### 2.2. Compound Isolation and Modification

**Figure 1:**
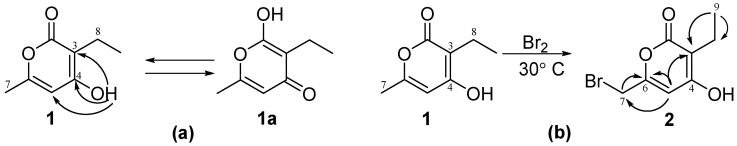
(**a**) Theoretical tautomeric equilibrium of **EHMP** and key HMBC correlations of **EHMP** in acetone-*d*_6_. (**b**) Scheme of the synthesis of the Br derivative (**2**) of **EHMP** (**1**) and key HMBC correlations in **Br-EHMP** (**2**).

The structure of isolated compound **1** (Figure 1) was established using NMR and MS data (Table 1, Figures S2–S5 and S15), which identified it as 3-ethyl-4-hydroxy-6-methyl-*2H*-pyran-2-one (**EHMP**, **1**). It should be noted that **EHMP** may theoretically exist in two tautomeric forms: α-pyrone (**1**) and γ-pyrone (**1a**) (Figure 1a). Usually, IR spectroscopy is used to determine the major tautomeric form [14]. Unfortunately, **EHMP** is not dissolved in CDCl_3_ or CCl_4_ (solvents without hydroxy and carbonyl groups); therefore, appropriate IR spectra cannot be obtained. Nevertheless, the NMR spectrum (Table S3, Figures S6–S9) obtained in acetone-*d*_6_ contained the signal of 4-OH (δ_H_ 9.82) and HMBC correlations (Figure 1a) from 4-OH to C-3 (δ_C_ 103.5), C-4 (δ_C_ 164.0), and C-5 (δ_C_ 99.6) undoubtedly proved an α-pyrone form of **EHMP**.

The bromination of compound **1** was carried out with Br_2_ at 30 °C for 2 h, as shown in Figure 1b. The separation of the reaction mixture yielded compound **2** (0.88 mg), **EHMP** (6.0 mg), and approximately 1 mg of an unidentified minor product mixture with a molecular formula of C_8_H_8_Br_2_O_3_ (Figure S17).

The ^1^H and ^13^C NMR spectra of compound **2** (Figure 1b) were similar to those of compound **1**, except for the proton and carbon signals at C-5, C-6, and CH_2_-Br (Table 1). The structure of compound **2** (Figure 1b) was established based on the NMR (Table 1, Figures S10–S14) and MS data (Figure S16) as 6-(bromomethyl)-3-ethyl-4-hydroxy-2H-pyran-2-one (**Br-EHMP**, **2**).

### 2.3. Antimicrobial Activity of Compounds

The effects of compounds **1** and **2** on the growth and biofilm formation of *Staphylococcus aureus*, *Escherichia coli*, and *Candida albicans* were investigated, and the data are presented in Table 2. The growth of the microbial cultures was detected using optical density measurements, and biofilm metabolic activity was studied using MTT labeling of formed biofilms, as described in the Section 4.

Compound **1** weakly inhibited the growth of all test strains and did not affect the formation of *S. aureus* and *E. coli* biofilms; however, it significantly prevented *C. albicans* biofilm formation, with an IC_50_ of 49.3 µM.

Compound **2** was more effective against *S. aureus* growth but less effective against C. albicans growth than compound **1**. In addition, compound **2** effectively inhibited the formation of *S. aureus* biofilms with an IC_50_ near 100 µM, but **2** was less effective against *C. albicans* biofilms. Both **1** and **2** exhibited weak activity against *E. coli*.

**Figure 2:**
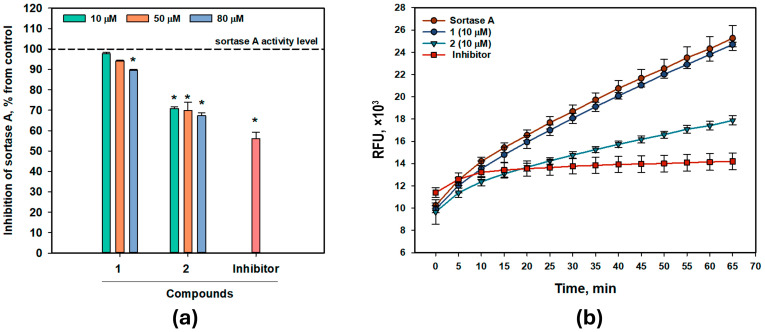
Influence of EHMP (**1**) and Br-EHMP (**2**) on sortase A activity. (**a**) Percentage of sortase A inhibition after 10 min of reaction. (**b**) Time-dependent graph of the inhibitory effect of **1** and **2** on sortase A activity. 4-(hydroxymercuri)benzoic acid (PCMB) was used as an inhibitor. Data are presented as mean ± SEM. All experiments were performed in triplicate. Statistical significance was evaluated using the *t*-test with SigmaPlot 14.0 software. * indicates significant differences between the experimental and control levels with a *p*-value < 0.05.

Because *S. aureus* biofilm formation is dependent on sortase A enzymatic activity [15], we investigated the inhibitory effects of **1** and **2** on sortase A activity (Figure 2) using a cell-free assay.

Unmodified compound **1,** only at 80 µM, significantly inhibited sortase A activity by 10.2%. Bromine-containing compound **2** at 10, 50, and 80 µM inhibited sortase A activity by 29.2%, 30.1%, and 32.7%, respectively. It should be noted that the IC_50_ was not achieved even with an increase in the concentration of **2**. The percentage of sortase A activity inhibition remained similar with an increase in concentration 5 and 8 times. This may indicate the saturation of the active site of the enzyme or other phenomena. We have already observed such an effect when studying the activity of dendryol B [16].

The interactions of compounds **1** and **2** with sortase A (PDB ID 1T2P) were studied using molecular docking and molecular dynamics simulations. The crystal structure of native sortase A from *Staphylococcus aureus,* deposited in the RCSB Protein Data Bank (https://www.rcsb.org, accessed on 1 December 2025) with PDB ID 1T2P, has only A, B, and C chains (total length 146 a.o.) without substrate [17]. When interacting with a substrate, the spatial structure of the enzyme changes; therefore, using a native structure is preferable if we want to analyze how an external ligand can interact with this enzyme. This structure has been used many times in molecular docking calculations [18,19], including by us [16], and the theoretical data correlate well with experimental data [20,21]. The active site of sortase A is formed by the β4, β7, and β8 strands on one side of the β-barrel, together with three surrounding loops on the other side. The left side of the pocket is a hydrophobic tunnel formed by Ala92, Ala104, Ala118, Val161, Pro163, Val166, Val168, Ile182, Val193, Trp194, Ile199, and Val201, along with two putative catalytic residues, Cys184 and Arg197. The right side of the pocket consists of several polar residues: Glu105, Asn114, Ser116, and Thr180 [17]. Arg197, Cys184, and His120 constitute the catalytic triad and are significant for sortase A enzyme activity.

**Figure 3:**
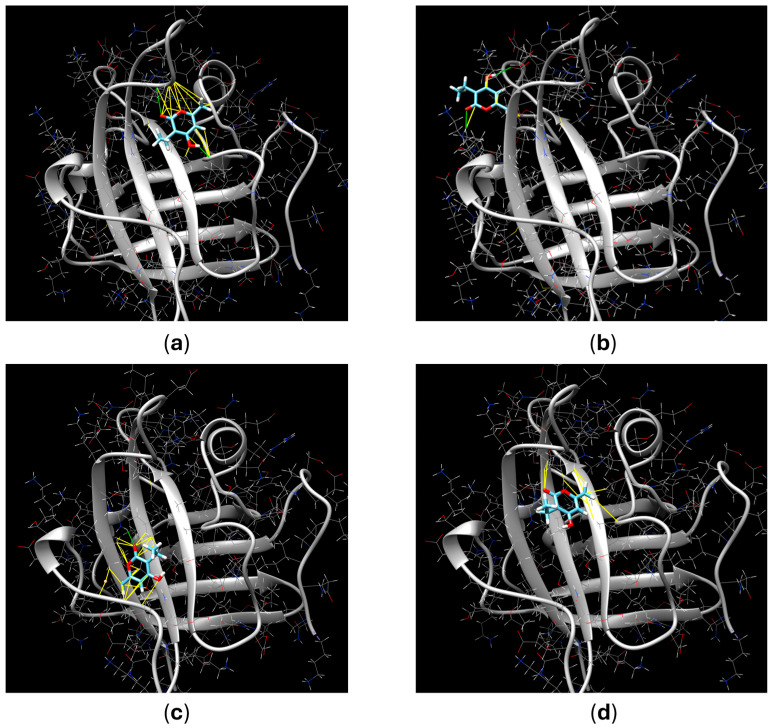
Molecular docking poses of sortase A (PDB ID 1T2P) complexes **1a** (**a**), **1b** (**b**), **2a** (**c**), and **2b** (**d**). The yellow and green lines represent hydrophobic and hydrogen-binding interactions, respectively. In the ligand structure, the light blue lines represent the carbon skeleton, protons are white, and oxygen atoms are red.

Thus, molecular docking revealed two more efficient complexes for each compound, and their characteristics are presented in Table 3 and visualized in Figure 3.

Briefly, compound **1** can form a binding pose with a ∆G of −6.26 kcal/mol, designated as pose **1a**, involving hydrogen bonds with Trp194 and Ala104, as well as hydrophobic interactions with Ala104, Gly192, Val193, and Trp194. Pose **1b**, with a ∆G of −6.40 kcal/mol, was calculated without hydrogen bonding interactions. Hydrophobic interactions at this position were calculated using Lys198, Lys196, and Thr183. Compound **2** can form pose **2a** with a ∆G of −6.72 kcal/mol and hydrogen-bonding interactions with Arg197 and hydrophobic interactions with Ile199, Leu169, Val168, and Ile182. Another pose, 2b, with a ∆G of −6.04 kcal/mol, was formed without hydrogen bonding and hydrophobic interactions with Ala104, Ala92, Trp194, Ala118, Val193, and Cys184 (Figure 3).

Thus, the enhanced inhibition of sortase A activity by **2** may be attributed to its interaction with Arg197 and Cys184 in the active site of the enzyme.

**Figure 4:**
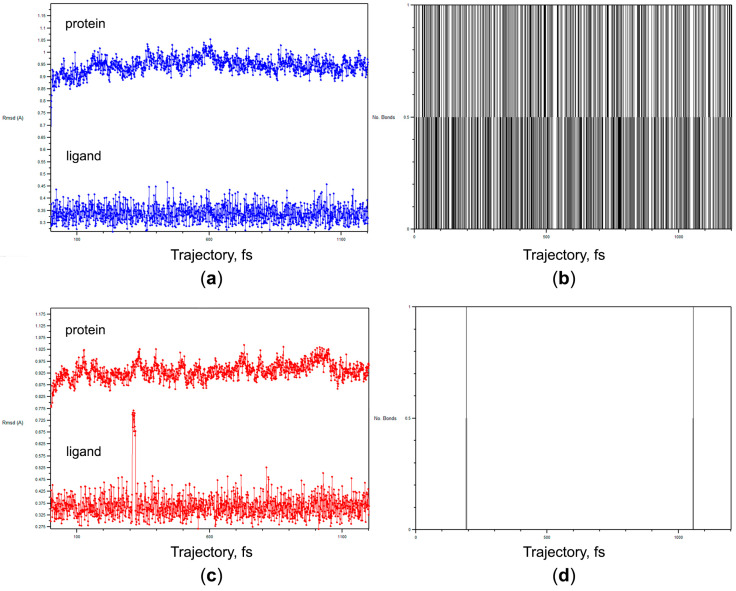
Analysis of the molecular dynamics simulations of the **1a** (**a**,**b**) and **1b** (**c**,**d**) complexes of **1** and sortase A.

**Figure 5:**
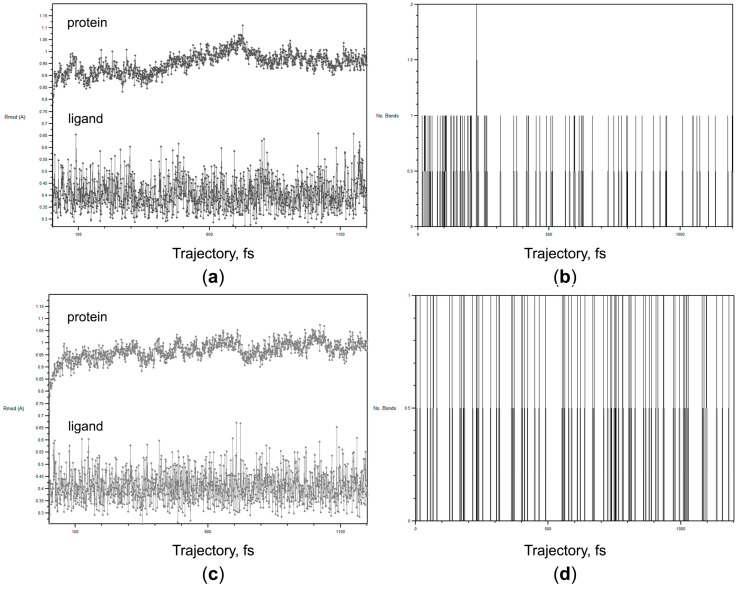
Analysis of the MD simulations of the **2a** (**a**,**b**) and **2b** (**c**,**d**) complexes of **2** and sortase A.

These predicted complexes were analyzed using a molecular dynamics (MD) simulation approach with a 12.5 ns trajectory. The root-mean-square deviation (RMSD) data and dynamics of the formed hydrogen binding were analyzed (Table 4, Figure 4 and Figure 5). RMSD was computed after the alignment of atomic coordinates in each trajectory step to a reference structure and indicates the average displacement of the atoms at an instant of the simulation relative to a reference structure [22]. The RMSD is used to discern whether a structure is stable on the time scale of the simulations, and an RMSD value of less than 3 indicates weak fluctuation. In our calculations, the protein RMSD was not more than 1.111 Å and the ligand RMSD was not more than 0.663 Å (Table 4), indicating the stability of all predicted complexes during the trajectories.

The complex of **1a** with sortase A had one hydrogen binding during the calculated trajectory (Figure 4b), whereas the complex of **1b** with sortase A had unstable hydrogen binding (Figure 4d). Simultaneously, the complex of **2a** with sortase A had one formed hydrogen binding and rarely a second hydrogen binding during the calculated trajectory (Figure 5b), and the complex of **2b** with sortase A had two stable hydrogen bindings (Figure 5d).

Thus, molecular docking and molecular dynamics simulation calculations were fully correlated with the experimental data. Both compounds **1** and **2** may interact with sortase A; however, only compound **2** may form a complex in the active site with Cys184, and these interactions result in the inhibition of sortase A activity. Of course, the calculated trajectories are small and insufficient for long-term predictions [23], but the experimental data that compound **2** continues to inhibit sortase A activity for 60 min (Figure 2b) is primary. However, regardless of the duration of the MD simulations, the results can only be confirmed experimentally.

### 2.4. Cytotoxic Activity of Compounds

Normal rat cardiomyocytes of the H9c2 line were used to study the cytotoxic activity of **1** and **2**. Compound **1** at 10 and 100 µM decreased the viability of H9c2 cells by 17.2 ± 2.3% and 35.8 ± 5.1%, respectively, and was nontoxic to H9c2 cells at 1 µM. Compound **2** at 100 µM decreased the viability of H9c2 cells by 48.9 ± 3.8% and was not toxic at 1 and 10 µM. All data were significantly different from the untreated cell viability data with a *p*-value less than 0.05.

The ADMET and QED properties of **EHMP** (**1**) and **Br-EHMP** (**2**) were predicted using the Syntelly platform, and the results are presented in Table S4. The in silico ADMET predictions indicate that **Br-EHMP** (**2**) may be more drug-like and potentially less toxic than **EHMP** (**1**), suggesting a favorable preliminary toxicological profile that warrants further investigation.

## 3. Discussion

α-pyrones are reported as phytotoxins produced by plant pathogenic fungi that interfere with plant physiological processes [24]. Three α-pyrones (fupyrones A and B, and 4-methyl-5,6-dihydro-*2H*-pyran-2-one) were isolated from *Fusarium* sp. and were active in antibacterial and quorum-sensing inhibition assays [25]. α-pyrones from *Aspergillus* fungi exhibit antimicrobial, anti-inflammatory, and anticancer effects [26]. Eight new α-pyrone derivatives from *Aspergillus niger* MA-132 exhibited weak cytotoxicity against several tumor cell lines [27].

We obtained 3-ethyl-4-hydroxy-6-methyl-*2H*-pyran-2-one (**EHMP**) from the marine holothurian-derived fungus *Trichoderma koningii* KMM 4751. Earlier, **EHMP** was isolated as a natural compound from a marine sponge-associated fungus, *Truncatella angustata* [28], terrestrial plant-derived fungi, *Trichoderma spirale* A725 [25], *Trichoderma* sp. MWTGP-04 [29], and *Raffaelea quercivora* [30]. Moreover, this compound is often obtained synthetically using various approaches [31,32,33].

Earlier, **EHMP** has been reported to be an antifungal agent [29]. In our experiments, **EHMP** weakly inhibited the growth of *Staphylococcus aureus*, *Escherichia coli*, and *Candida albicans* strains and affected the formation of *C. albicans* biofilms. Synthetic modification of the **EHMP** structure via bromination significantly increased the anti-biofilm anti-staphylococcal properties of **Br-EHMP** without enhancing toxicity to cardiomyocytes H9c2.

*S. aureus* biofilms play a significant role in the development of chronically infected wounds [34], pneumonia, vascular infections, and implant infections. Biofilm formation is controlled by the *quorum-sensing* system and several extracellular enzymes, including sortases A and B [35], whereas the inhibition of their activities can prevent or disrupt biofilm formation. Previously, we reported that the marine fungal tripeptide asterripeptide C prevents *S. aureus* biofilm formation by inhibiting sortase A activity, enhances wound healing in vivo, and protects mice from sepsis [36].

Bromine-containing compounds can be even more effective as anti-staphylococcal agents. More pronounced antimicrobial and antibiotic properties of brominated compounds compared to non-brominated compounds have been demonstrated [4]. 2,7-dibromophenanthrenequinone and 3,6-dibromophenanthrenequinone disrupted *Staphylococcus aureus* biofilms more effectively than their non-brominated counterparts [5]. The bromine atom in N-(2-bromo-4,4-dimethyl-3-oxocyclobut-1-en-1-yl)-N-methylbenzamide was found to be crucial for binding to Cys115 in another bacterial target, UDP-N-acetylglucosamine enolpyruvyl transferase (MurA) [37]. The authors of this study confirmed the specific covalent interactions between Br and Cys115 and the formation of stable adducts. In our molecular docking calculations, only **Br-EHMP** interacted with the crucial Cys184 in the active site of sortase A. While the mechanism of interaction between Br-EHMP and the active site of sortase A requires experimental validation, our calculations suggest a potentially stable interaction between Br in **Br-EHMP** and Cys184. This may explain why the inhibition of sortase A activity occurred at the same level under the action of different concentrations of **Br-EHMP**: a stable interaction can form at a low concentration of a compound, and an increase in concentration will not lead to an increase in activity. Our data confirm that the strategy of choosing brominated compounds, including the bromination of natural compounds, as potential anti-biofilm leading molecules is feasible.

## 4. Materials and Methods

### 4.1. General Experimental Procedures

^1^H and ^13^C NMR spectra were recorded in aceton-*d*_6_ on Bruker Avance-300 and Avance-500 spectrometers (Bruker BioSpin GmbH, Bremen, Germany) operating at 300 and 75, 500, and 125 MHz, respectively. HRESIMS spectra were obtained using a Bruker maXis Impact II mass spectrometer (Bruker Daltonics GmbH, Bremen, Germany). Low-pressure liquid column chromatography was performed using silica gel (50/100 μm; Imid Ltd., Krasnodar, Russia). Plates pre-coated with silica gel (5–17 μm, 10 cm × 10 cm, Imid Ltd., Krasnodar, Russia) and 60 RP-18 F254S silica gel (20 cm × 20 cm, Merck KGaA, Darmstadt, Germany) were used for thin-layer chromatography. Preparative HPLC was performed on an Agilent 1100 chromatograph (Agilent Technologies, Santa Clara, CA, USA) with an Agilent 1100 refractometer (Agilent Technologies, Santa Clara, CA, USA) and a Shimadzu LC-20 chromatograph (Shimadzu USA Manufacturing, Canby, OR, USA) with a Shimadzu RID-20A refractometer (Shimadzu Corporation, Kyoto, Japan) using semi-preparative columns YMC ODS-AM (YMC Co., Ishikawa, Japan) (5 µm, 10 mm × 250 mm), HyperClone ODS (C-18) (Phenomenex, 5 µm, 4.6 mm × 250 mm, Torrance, CA, USA), YMS-Pack SIL (YMC Co., Ishikawa, Japan) (5 µm, 10 mm × 250 mm), and Nautilus 110-5-C18 R (BioKhimMak ST, Moscow, Russia) (5 µm, 10 mm × 250 mm) columns.

### 4.2. Fungal Strain and Culture

The marine fungal strain KMM 4751 was isolated from the Far Eastern sea cucumber *Apostichopus japonicus* collected from Peter the Great Bay (Sea of Japan) and identified as *Trichoderma koningii* based on its morphological features. The strain was stored in the Collection of Marine Microorganisms of G.B. Elyakov Pacific Institute of Bioorganic Chemistry (Vladivostok, Russia).

### 4.3. Cultivation of Fungus

Analytical cultivation of the KMM 4751 fungus was conducted at room temperature for 14 and 21 days on rice medium (one 500 mL Erlenmeyer flask), malt extract agar (two Petri dishes), and wort agar (two Petri dishes). The composition of the culture media is provided in Table S1.

Scaled-up cultivation of the KMM 4751 fungus for metabolite isolation was conducted for 21 days on rice medium in 100 Erlenmeyer flasks (500 mL).

### 4.4. Isolation and Structure Elucidation of 1

At the end of the incubation period, the mycelia and medium were extracted with EtOAc, the extract was concentrated and fractionated as described earlier [38]. The fraction eluted with an EtOAc–hexane (20:80) solution was purified by HPLC on a Synergi Fusion-RP column in a MeOH–H2O–TFA (90:10:0.1) to obtain compound **1**.

Compound **1** is a white amorphous powder, C_8_H_10_O_3_, *m*/*z*: 153.0561 [M−H]^−^ (calcd. 153.0557 for C_8_H_9_O_3_), 155.0703 [M+H]^+^ (calcd. 155.0703 for C_8_H_11_O_3_). The ^1^H NMR spectrum (700 MHz, methanol-d_4_, *δ*, ppm, *J*, Hz): 1.0 (3H, t, *J* = 7.4, CH_3_), 2.2 (3H, s, CH_3_), 2.4 (2H, q, *J* = 14.8, 7.4, CH_2_), 6.0 (1H, s, H-5). The ^13^C NMR spectrum (176 MHz, methanol-d_4_, *δ*, ppm): 168.2 (C-4), 167.5 (C-2), 161.7 (C-6), 105.0 (C-3), 101.6 (C-5), 19.5 (C-7), 17.2 (C-9), 12.8 (C-8).

### 4.5. Obtaining the Brominated Derivative 2

A solution of compound **1** (10 mg) in 0.6 mL CDCl_3_ was treated dropwise with a solution of Br_2_ (0.4 mg) in CDCl_3_ at 30 °C, monitoring the reaction progress by NMR. After 2 h, the solvent was evaporated, and the residue was purified by HPLC on a YMC Chiral column eluted with 50% MeOH and 0.1% TFA to afford 0.88 mg of compound **2** (yield: 5.82%).

Compound **2** is a white amorphous powder, C_8_H_9_BrO_3_, *m*/*z*: 230.9663 [M−H]^−^ (calcd. 230.9662 for C_8_H_8_BrO_3_), 232.9663 [M+H]^+^ (calcd. 232.9663 for C_8_H_10_BrO_3_). The ^1^H NMR spectrum (700 MHz, methanol-d_4_, *δ*, ppm, *J*, Hz): 1.0 (3H, t, *J* = 7.4, CH_3_), 2.4 (2H, q, *J* = 14.8, 7.3, CH_2_), 4.3 (2H, s, CH_2_-Br), 6.3 (1H, s, H-5). The ^13^C NMR spectrum (176 MHz, methanol-d_4_, *δ*, ppm): 166.0 (C-2), 165.0 (C-4), 157.0 (C-6), 106.3 (C-3), 101.9 (C-5), 26.4 (C-7), 16.1 (C-8), 11.2 (C-9).

### 4.6. Strains and Antimicrobial Assay

The Gram-positive bacterium *Staphylococcus aureus* ATCC 21027, the Gram-negative bacterium *Escherichia coli* VKPM (B-7935), and the yeast-like fungus *Candida albicans* KMM 455 strains were cultured on solid medium Mueller Hinton broth with agar (16.0 g/L) in Petri dishes at 37 °C for 24 h.

The compounds were dissolved in DMSO at a stock concentration of 10 mM and then tested at concentrations ranging from 100 µM to 1.563 µM through serial two-fold dilutions in 96-well plates. The effect of the compound on the growth of *S. aureus*, *E. coli*, and *C. albicans* was estimated according to [39]. The optical density of the microbial suspension after 18 h of treatment was measured at λ = 620 nm.

The effect of the compounds on biofilm formation for 18 h was tested using MTT reagent (Sigma-Aldrich, St. Louis, MO, USA) [40]. The microbial suspension was gently removed from the wells, and the formed biofilms were washed with sterile PBS. Then, the MTT reagent solution was added to each well for 2 h. After that, the biofilm was lysed using DMSO (100%). The optical density of the obtained solution was measured at λ = 570 nm.

Gentamicin (1 mg/mL) was used as a positive control for *S. aureus* and *E. coli* tests. Nitrofungin (1 mg/mL) was used as a positive control for the *C. albicans* test. DMSO at a 1% solution in phosphate-buffered saline (PBS) was used as a negative control. A Multiskan FS spectrophotometer (Thermo Scientific Inc., Beverly, MA, USA) was used for both assays.

The results of both assays were expressed as percentages of the control data, and IC_50_s and the percentages of growth or biofilm formation inhibition were calculated.

### 4.7. Sortase A Activity Inhibition Assay

The enzymatic activity of sortase A from *Staphylococcus aureus* was determined using a SensoLyte 520 Sortase A Activity Assay Kit Fluorimetric (AnaSpec AS-72229, Ana-Spec, San Jose, CA, USA). 4-(hydroxymercuri)benzoic acid (PCMB) was used as a sortase A enzyme activity inhibitor. The PCMB stock solution concentration was 10 mM, and the concentration in the reaction mixture was 20 µM. Fluorescence was measured using a PHERAStar FS plate reader (BMG Labtech, Offenburg, Germany) for 60 min at a time interval of 5 min, with λ_ex_ = 490 nm and λ_em_ = 520 nm. The data were processed using MARS Data Analysis v. 3.01R2 (BMG Labtech, Offenburg, Germany).

### 4.8. Molecular Docking and Molecular Dynamics Simulation

The PDB file of sortase A (PDB ID: 1T2P) was obtained from the RCSB Protein Data Bank (https://www.rcsb.org) and prepared for docking using the PrepDock package of UCSF Chimera 1.16 software. The chemical structures of the compounds were prepared for docking using Chem3D 17.0 (ChemOffice package) and verified using the PrepDock package of UCSF Chimera 1.16 software. Docking was conducted on the SwissDock online server (http://www.swissdock.ch, access date: 10 October 2025) using the EADock DSS docking software [41]. The algorithm implies the generation of many binding modes in the vicinity of all target cavities (blind docking) and the estimation of their CHARMM energies [42] for the evaluation of the binding modes with the most favorable energies from the Fast Analytical Continuum Treatment of Solvation (FACTS) [43], and finally, the clustering of these binding modes [44]. The predicted building models for the compound/sortase A pair were analyzed using UCFS Chimera 1.16 software. Docking parameters, including Gibbs free energy (ΔG, kcal/mol), hydrogen bonding, and hydrophobic interactions, were used to analyze the target/ligand complexes [45].

The best-predicted complexes were analyzed using MD simulations in GROMACS (v. 2023.3). The download file for the calculations was prepared using the CHARMM-GUI (https://www.charmm-gui.org, access date: 17 October 2025). The basic conditions of the solution were selected for the calculations. The simulation of molecular dynamics in GROMACS was carried out in steps of 0.002 ps, and coordinates were recorded every 5000 steps. The trajectory length was 12.5 ns. The analysis of the trajectories obtained was carried out using the VMD 2.0.0a7 program.

### 4.9. Cell Line, Culture Conditions, and MTT Assay

A detailed description of cell cultivation conditions was reported earlier [38].

### 4.10. Statistical Data Evaluation

A statistical data evaluation was performed as described earlier [38].

## 5. Conclusions

Thus, 3-ethyl-4-hydroxy-6-methyl-*2H*-pyran-2-one (**EHMP**) was isolated from the sea cucumber-associated fungus *Trichoderma koningii* KMM 4751, and its previously unreported brominated derivative, 6-(bromomethyl)-3-ethyl-4-hydroxy-*2H*-pyran-2-one (**Br-EHMP**), was obtained. **Br-EHMP** significantly inhibited the biofilm formation of *Staphylococcus aureus* and had a weak cytotoxicity to normal mammalian cells. Taken together, these activities make Br-EHMP an interesting anti-biofilm agent for future investigations, and bromination appears to be a viable approach for enhancing the antimicrobial properties of natural compounds.

## Figures and Tables

**Table 1:** The ^1^H and ^13^C NMR data (700 MHz, methanol-d_4_) for **1** and **2**.

**1**			**2**			
Position	δ_C_, mult	δ_H_ (*J* in Hz)	Position	δ_C_, mult	δ_H_ (*J* in Hz)	HMBC
2	168.6, C	-	2	167.4, C	-	-
3	105.0, C	-	3	107.7, C	-	-
4	167.5, C	-	4	166.4, C	-	-
5	101.6, CH	5.98, s	5	103.4, CH	6.29, s	3, 6, 7
6	161.7, C	-	6	158.5, C	-	-
7	19.5, CH_3_	2.19, s	7	27.8, CH_2_	4.28, s	5, 6
8	17.2, CH_2_	2.39, q (7.4)	8	17.5, CH_2_	2.42, q (7.3)	9
9	12.8, CH_3_	1.03, t (7.4)	9	12.6, CH_3_	1.04, t (7.4)	3, 8

**Table 2:** The effect of compounds on the growth and biofilm formation of test microorganisms ^1^.

Comp. ^2^	Growth Inhibition, %			Biofilm Formation Inhibition, %		
	*S. aureus*	*E. coli*	*C. albicans*	*S. aureus*	*E. coli*	*C. albicans*
**1**	18.93 ± 2.00 *	19.43 ± 1.47 *	43.04 ± 3.51 *	-	-	55.76 ± 2.13 *
**2**	45.28 ± 4.56 *	11.14 ± 1.04 *	4.29 ± 0.59	17.17 ± 1.15 *	-	28.89 ± 2.14 *

^1^ The data are presented as mean ± SEM. * indicates significant differences between the experimental and control groups at a *p*-value < 0.05. ^2^ Compounds were used at a concentration of 100 µM.

**Table 3:** Molecular docking calculations for sortase A complexes with **1** and **2**.

Complex	∆G, kcal/mol	Energy, kcal/mol	Hydrogen Binding, Å	Hydrophobic Interactions
**1a**	−6.2694707	0.543155	Trp194 … O1, 2.103 H6 … Ala104, 2.603	Ala104, Gly192, Val193, Trp194
**1b**	−6.400336	1.02235	Lys198 … O1, 2.279 H6 … Asp185, 2.153	Lys198, Lys196, Thr183
**2a**	−6.72635	−1.05381	Arg197 … O1, 2.095	Ile199, Leu169, Val168, Ile182
**2b**	−6.047532	2.99012	-	Ala104, Ala92, Trp194, Ala118, Val193, Cys184 … Br (3.732 Å)

**Table 4:** Molecular dynamic simulation data of sortase A complexes with **1** and **2**.

Complex	Protein RMSD, Å			Ligand RMSD, Å		
	Avg ± SD	Min	Max	Avg ± SD	Min	Max
**1a**	0.946 ± 0.038	0.263	1.054	0.171 ± 0.041	0.081	0.328
**1b**	0.933 ± 0.041	0.232	1.044	0.188 ± 0.064	0.073	0.663
**2a**	0.952 ± 0.048	0.256	1.111	0.244 ± 0.078	0.081	0.552
**2b**	0.967 ± 0.044	0.256	1.075	0.270 ± 0.075	0.088	0.584

## Data Availability

Data is contained within the article or Supplementary Materials.

## Supplementary Materials

The following supporting information can be downloaded at: https://www.mdpi.com/article/10.3390/antibiotics15060554/s1. Table S1. The cultivation media composition; Table S2. The extract yield depends on the cultivation time; Figure S1. TLC of various extracts of the marine fungus *Trichoderma koningi* KMM 4751, developed with an alcoholic solution of sulfuric acid (A), at 395 nm (B), and iodine vapor (C); Table S3. The 1H and 13C NMR data (300 MHz, acetone-d_6_) for **1**; Figure S2. ^1^H NMR spectrum (700 MHz, methanol-d_4_) of **1**; Figure S3. ^13^C NMR spectrum (176 MHz, methanol-d_4_) of **1**; Figure S4. HSQC spectrum (700 MHz, methanol-d_4_) of **1;** Figure S5. HMBC spectrum (700 MHz, methanol-d_4_) of **1**; Figure S6. ^1^H NMR spectrum (300 MHz, acetone-d_6_) of **1**; Figure S7. ^13^C NMR spectrum (300 MHz, acetone-d_6_) of **1**; Figure S8. HSQC spectrum (300 MHz, acetone-d_6_) of **1**; Figure S9. HMBC spectrum (300 MHz, acetone-d_6_) of **1**; Figure S10. ^1^H NMR spectrum (700 MHz, methanol-d_4_) of **2**; Figure S11. ^13^C NMR spectrum (700 MHz, methanol-d_4_) of **2**; Figure S12. DEPT-135 spectrum (700 MHz, methanol-d_4_) of **2**; Figure S13. HMBC spectrum (700 MHz, methanol-d_4_) of **2**; Figure S14. HSQC spectrum (700 MHz, methanol-d_4_) of **2**;. Figure S15: HR (–)ESI MS spectrum of **1**; Figure S16: HR (–)ESI MS spectrum of **2**; Figure S17: HR (–)ESI MS spectrum of mixture of unidentified products of bromination reaction; Table S4. ADMET properties of EHMP (**1**) and Br-EHMP (**2**).
