# Supplementary material for: Isolation, Bromination, and Antimicrobial Activity of 3-Ethyl-4-hydroxy-6-methyl-2H-pyran-2-one from Sea Cucumber-Associated Fungus Trichoderma koningii KMM 4751
Source: Antibiotics (Basel). 2026 May 30;15(6):554. doi: 10.3390/antibiotics15060554 (PMC13296077; doi:10.3390/antibiotics15060554)
Supplement: Supplementary file 1 [file antibiotics-15-00554-s001.zip › antibiotics-4288010-supplementary.pdf]

SUPPLEMENTARY MATERIAL

Isolation, Bromination, and Antimicrobial Activity of 3-Ethyl-4-hydroxy-6-methyl-2*H*-pyran-2-one from Sea Cucumber-Associated Fungus *Trichoderma koningii* KMM 4751

Content

Table S1. The cultivation media composition 3

Table S2. The extract yield depends on the cultivation time 3

Figure S1. TLC of various extracts of the marine fungus *Trichoderma koningi* KMM 4751, developed with an alcoholic solution of sulfuric acid (A), at 395 nm (B), and iodine vapor (C). 4

Table S3. The ^{1}H and ^{13}C NMR data (300 MHz, acetone-d_6) for 1 5

Figure S2. ^{1}H NMR spectrum (700 MHz, methanol-d_4) of 1 6

Figure S3. ^{13}C NMR spectrum (176 MHz, methanol-d_4) of 1 7

Figure S4. HSQC spectrum (700 MHz, methanol-d_4) of 1 8

Figure S5. HMBC spectrum (700 MHz, methanol-d_4) of 1 9

Figure S6. ^{1}H NMR spectrum (300 MHz, acetone-d_6) of 1 10

Figure S7. ^{13}C NMR spectrum (300 MHz, acetone-d_6) of 1 11

Figure S8. HSQC spectrum (300 MHz, acetone-d_6) of 1 12

Figure S9. HMBC spectrum (300 MHz, acetone-d_6) of 1 13

Figure S10. ^{1}H NMR spectrum (700 MHz, methanol -d_4) of 2 14

Figure S11. ^{13}C NMR spectrum (700 MHz, methanol-d_4) of 2 15

Figure S12. DEPT-135 spectrum (700 MHz, methanol-d_4) of 2 16

Figure S13. HMBC spectrum (700 MHz, methanol -d_4) of 2 17

Figure S14. HSQC spectrum (700 MHz, methanol-d4) of 2 18

Figure S15. HR (–)ESI MS spectrum of 1 19

Figure S16. HR (–)ESI MS spectrum of 2 19

Figure S17. HR (–)ESI MS spectrum of mixture of unidentified products of bromination reaction 20

Table S4. ADMET properties of EHMP (1) and Br-EHMP (2) 21

Table S1. The cultivation media composition

Cultivation media	Composition
Rice medium	Per flask: rice (20.0 g), yeast extract (20.0 mg), KH_2PO_4 (10 mg), natural sea water (40 ml) from Vodolaznaya bay, Troitsa bay, the Sea of Japan
malt extract agar (MEA)	Per liter: wort agar (Himedia, India) 48.28 g, glycerol (2.35 g), natural sea water (1000 ml) from Vodolaznaya bay, Troitsa bay, the Sea of Japan
wort agar (WA)	Per liter: Malt Extract Powder ((Himedia, India) 30 g, peptone (5 g), $CuSO_4$ (5 mg), $ZnSO_4$ (1 mg), natural sea water (1000 ml) from Vodolaznaya bay, Troitsa bay, the Sea of Japan

Table S2. The extract yield depends on the cultivation time

**Cultivation medium**	**Cultivation time, days**	**Amount of extract per 1 kg of medium, mg/kg**
Rice	14	14.7×10^{-4}
Rice	21	20.5×10^{-4}
Wort agar	14	2.4×10^{-4}
Wort agar	21	1.3×10^{-4}
Malt extract agar (MEA)	14	0.8×10^{-4}
Malt extract agar (MEA)	21	1.6×10^{-4}

Figure S1. TLC of various extracts of the marine fungus *Trichoderma koningi* KMM 4751, developed with an alcoholic solution of sulfuric acid (A), at 395 nm (B), and iodine vapor (C).

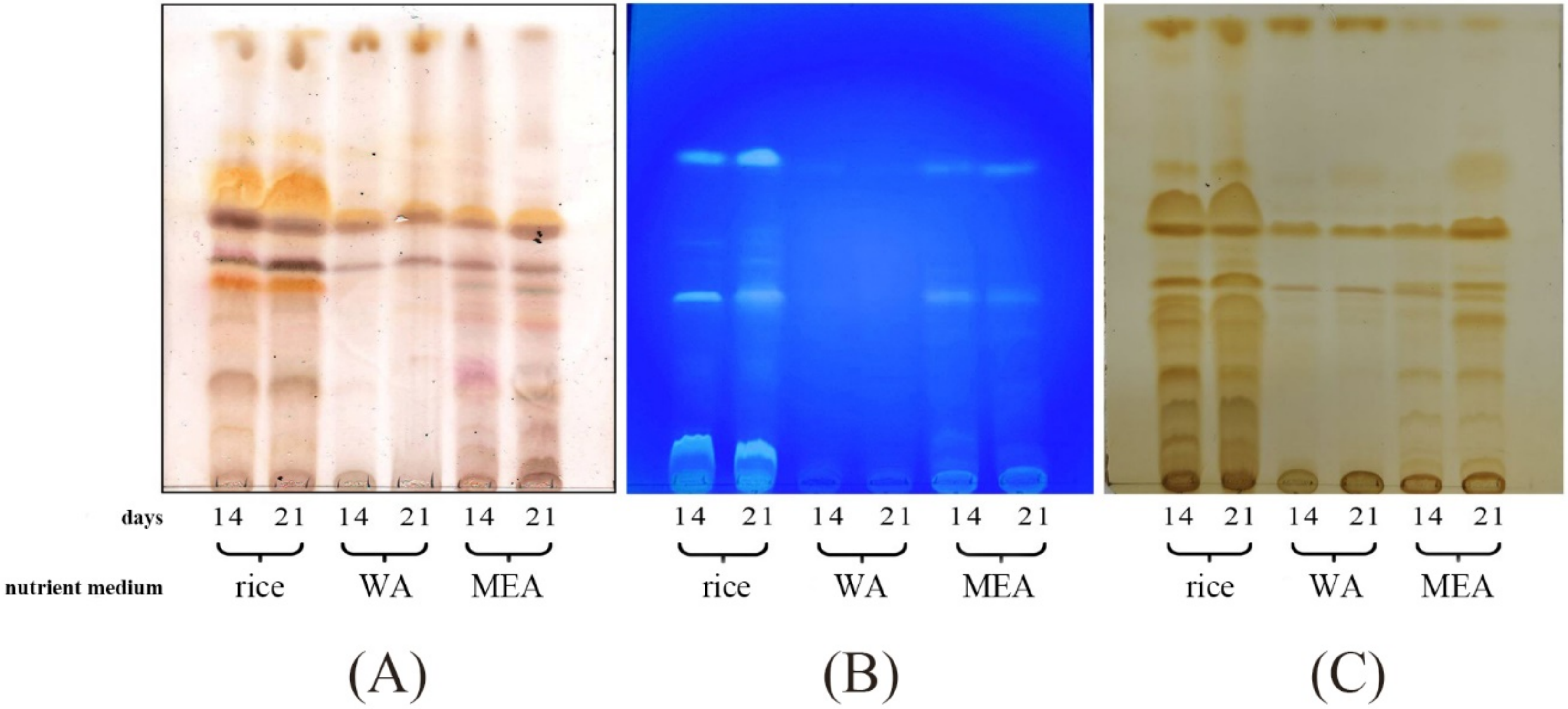

Analytical cultivation of the fungus was conducted to select the most suitable nutrient medium and optimal cultivation time. The composition of secondary metabolites in extracts obtained after cultivation on rice medium, malt extract agar (MEA), and wort agar (WA) for 14 and 21 days was analyzed (Fig. 2). Cultivation on rice medium resulted in higher yields of secondary metabolites. The qualitative composition of metabolites on day 21 of cultivation was virtually identical to that on day 14, while the total yield of metabolites increased on day 21 – the highest mass of extract was obtained after 21 days of cultivation on rice medium.

Table S3. The ^{1}H and ^{13}C NMR data (300 MHz, acetone-d_6) for 1

Posotion	δ_C, mult	δ_H (*J* in Hz)	HMBC
2	164.7, C	–	–
3	103.5, C	–	–
4	164.0, C	–	–
5	99.6, CH	6.00, s	3, 4, 6, 7
6	159.8, C	–	–
7	18.6, CH_3	2.14, s	5, 6
8	16.1, CH_2	2.39, q (7.4)	2, 3, 4, 9
9	11.9, CH_3	1.01, t (7.4)	3, 8
4-OH		9.82, s	3, 4, 5

Figure S2. ¹H NMR spectrum (700 MHz, methanol-d₄) of 1

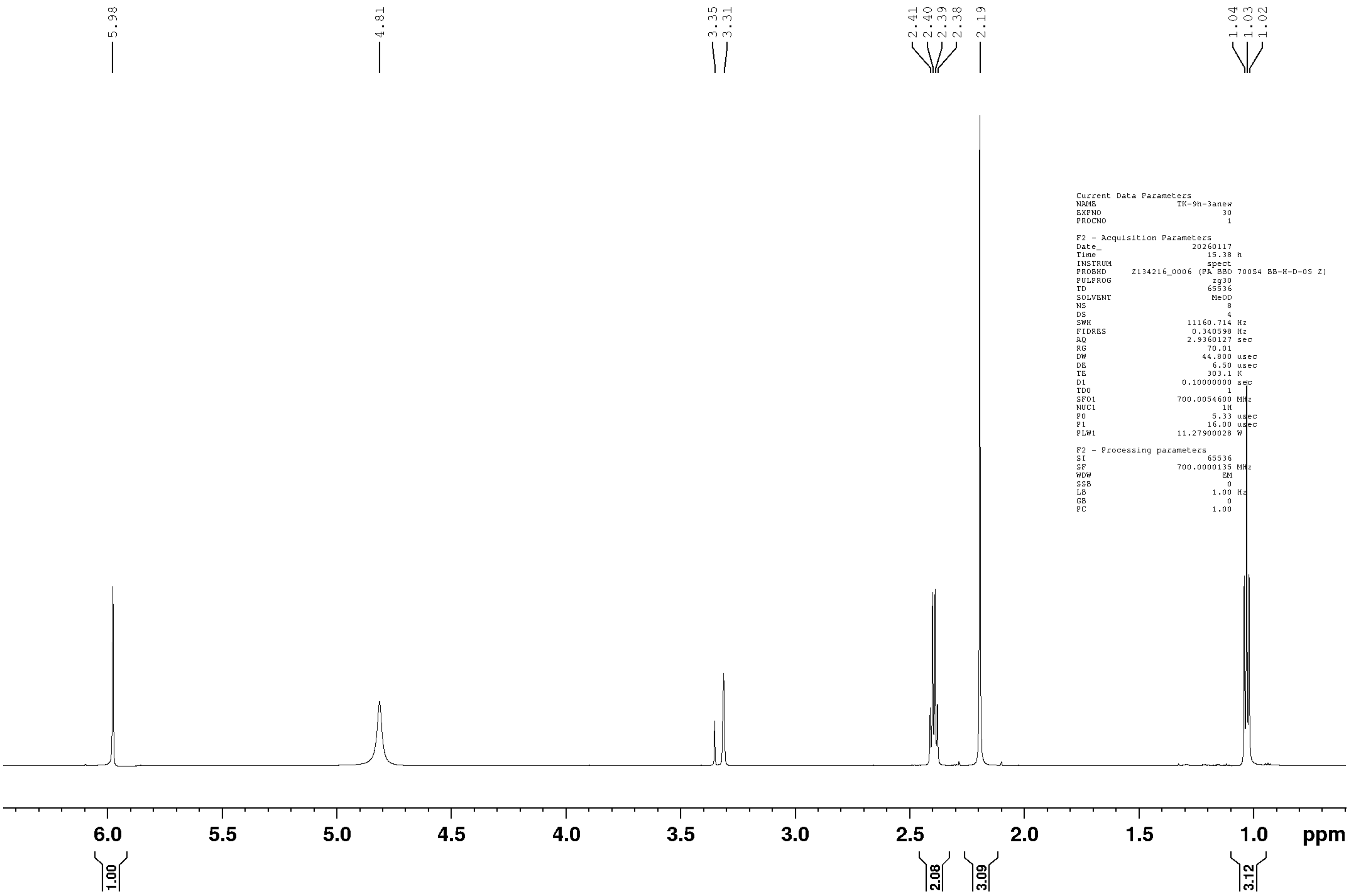

Figure S3. ^{13}C NMR spectrum (176 MHz, methanol-d_4) of 1

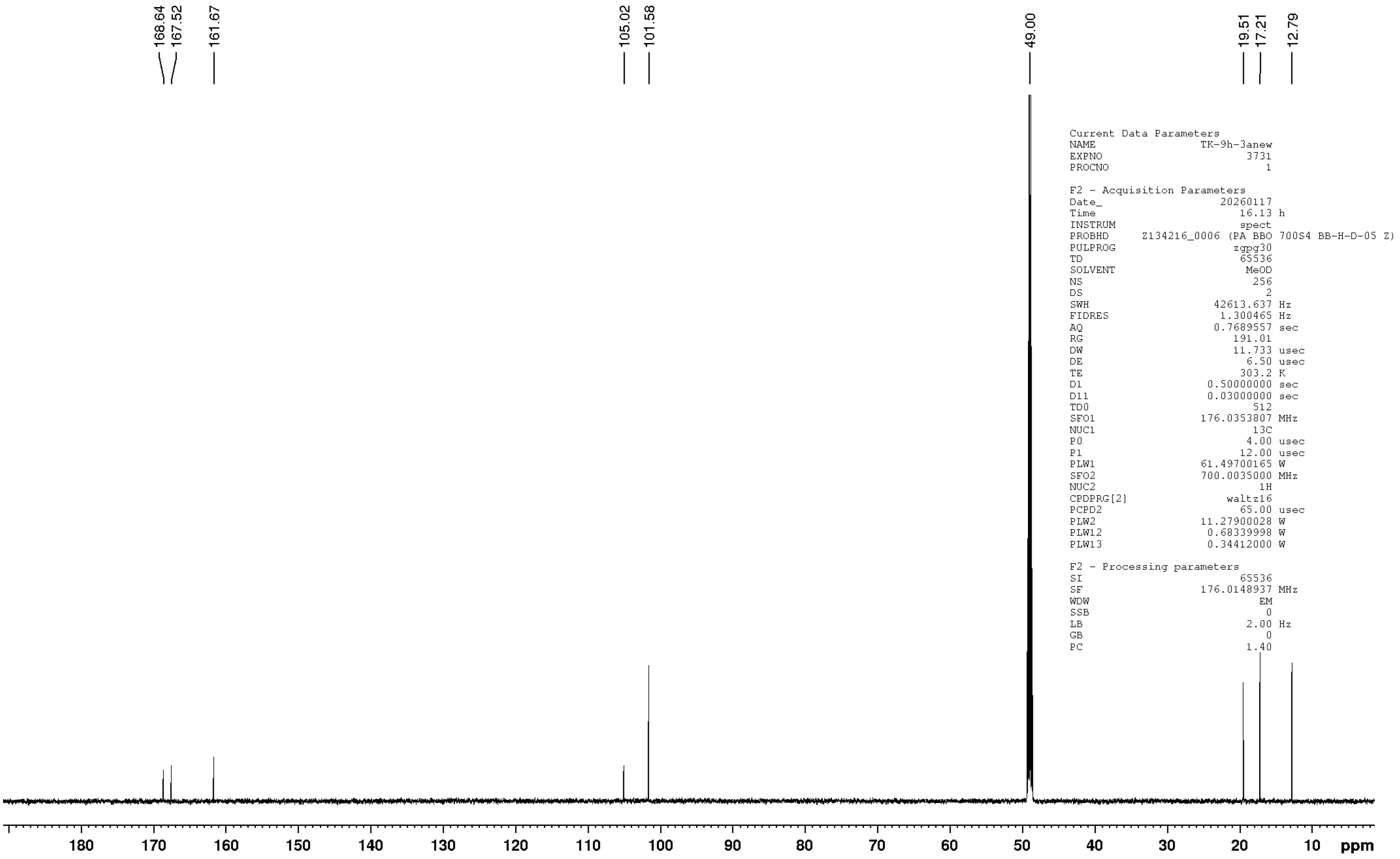

Figure S4. HSQC spectrum (700 MHz, methanol-d₄) of 1

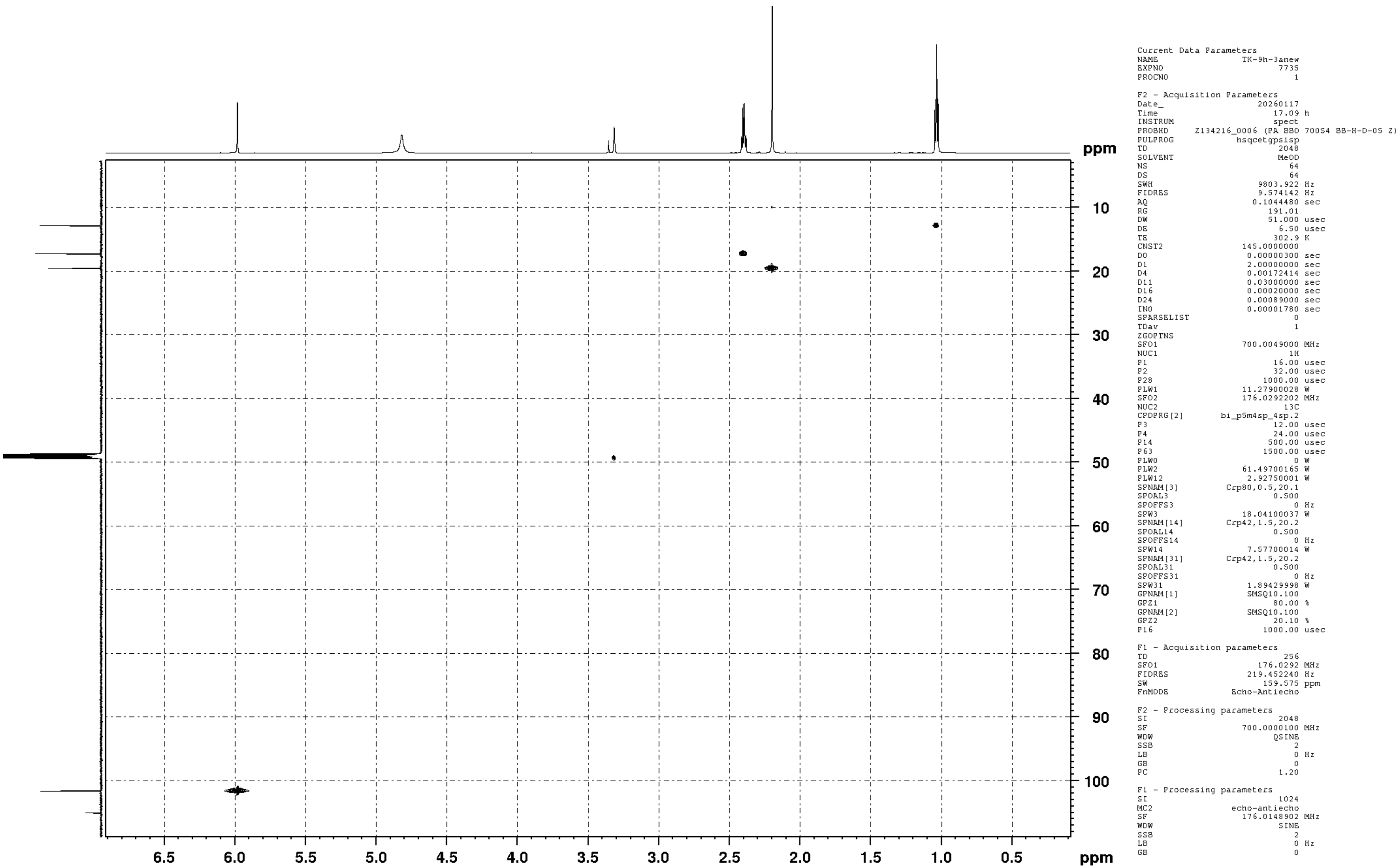

Figure S5. HMBC spectrum (700 MHz, methanol-d$_4$) of 1

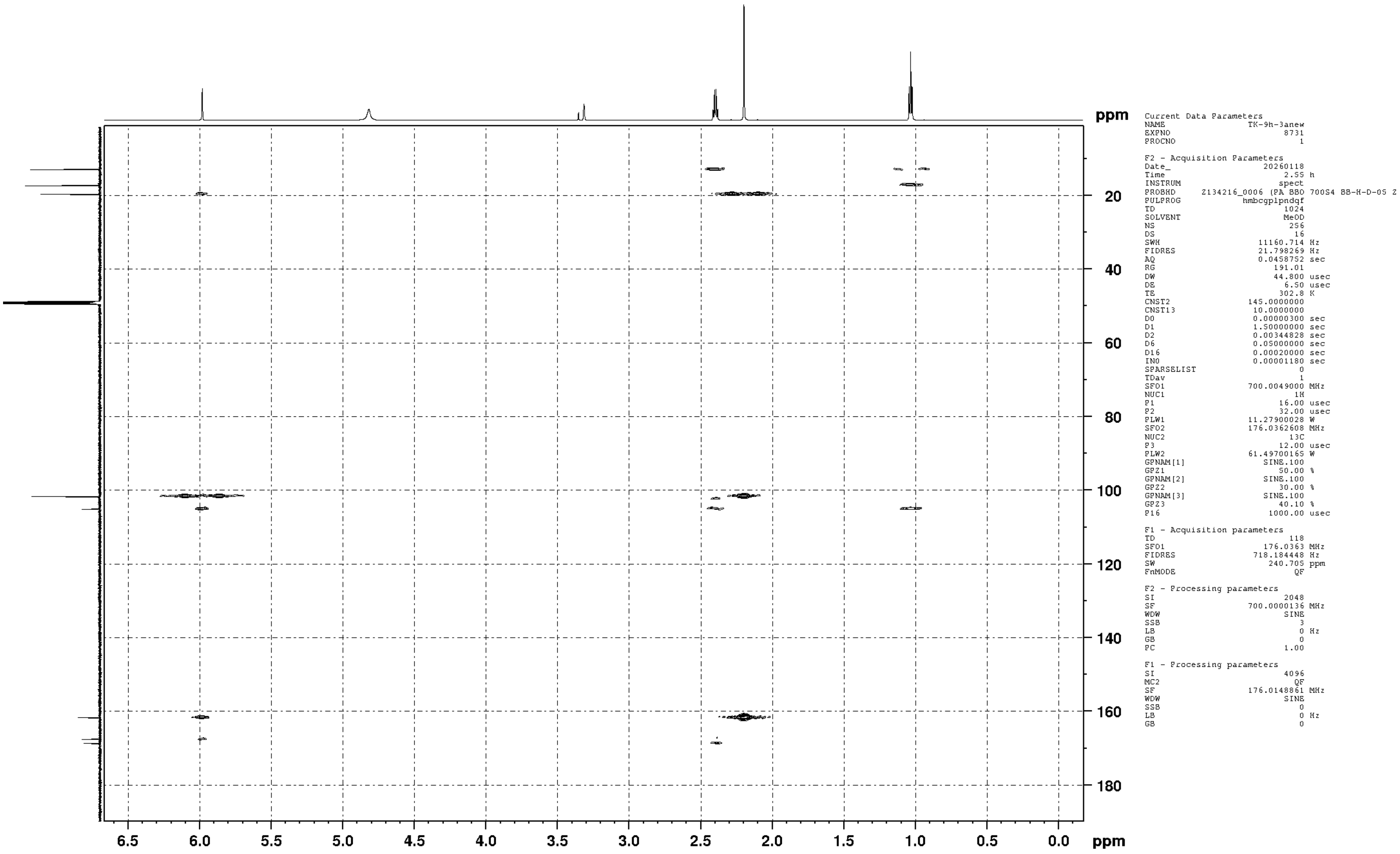

Figure S6. ^{1}H NMR spectrum (300 MHz, acetone-d_6) of 1

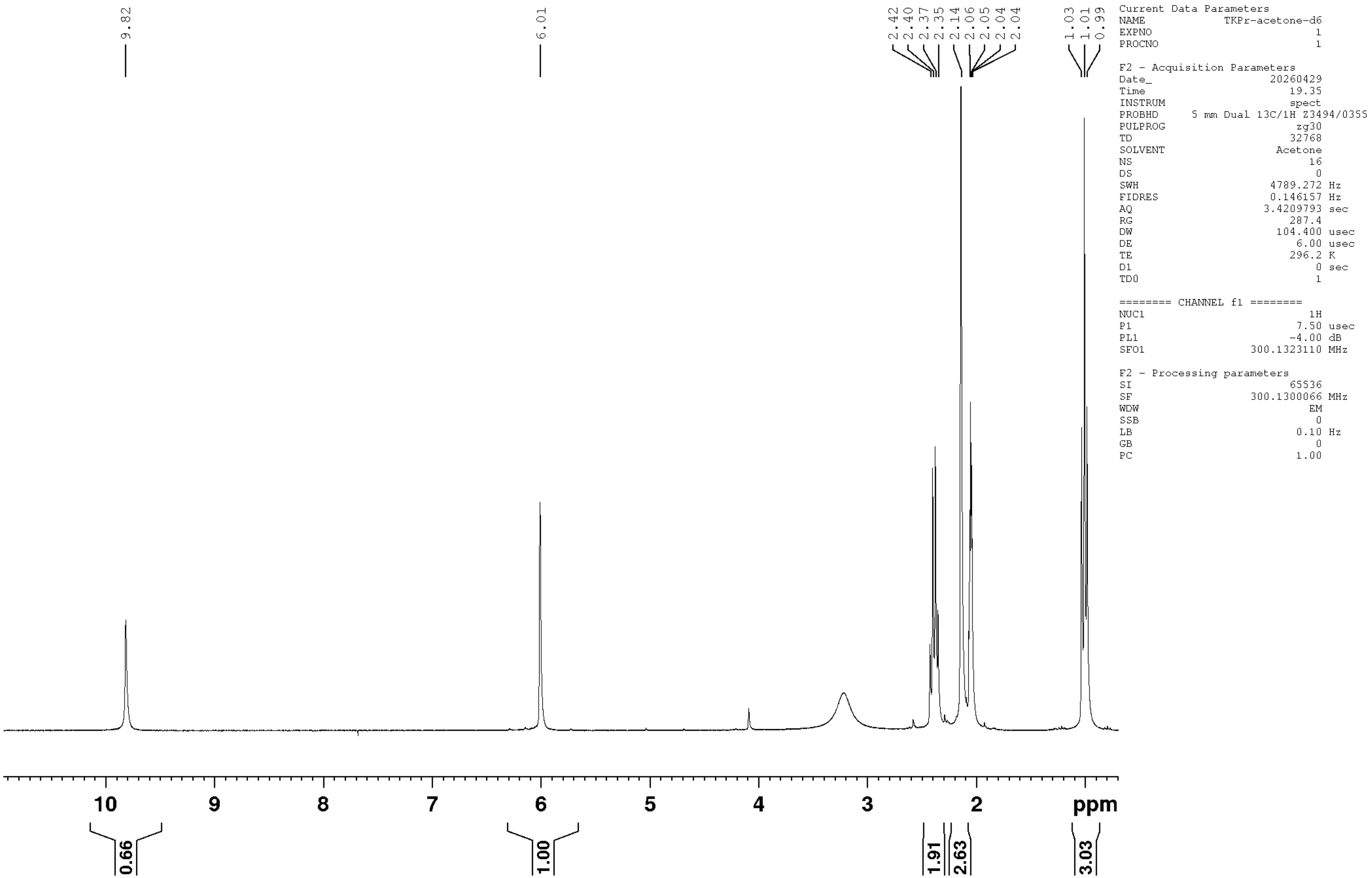

Figure S7. ^{13}C NMR spectrum (300 MHz, acetone-d6) of 1

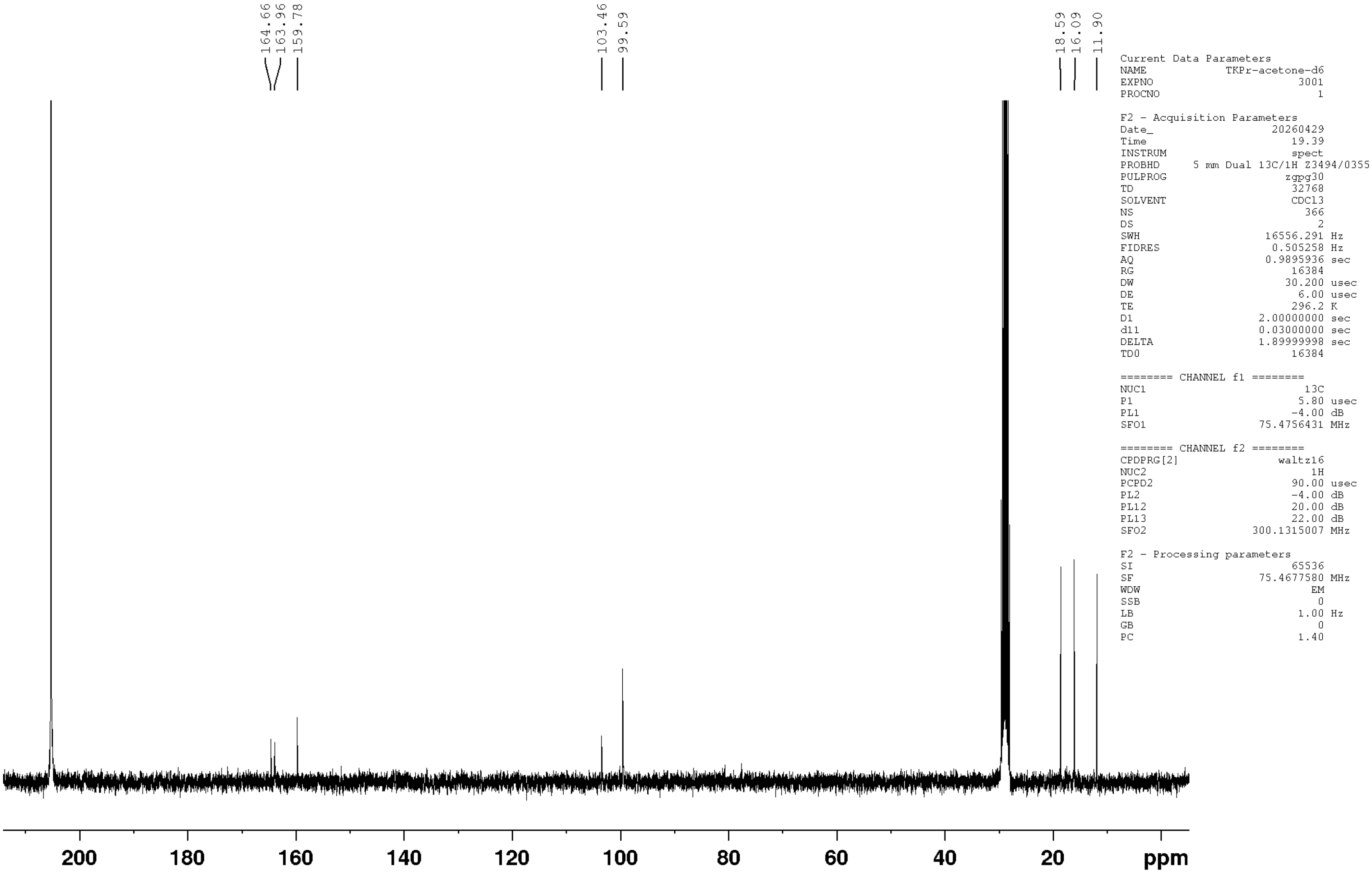

Figure S8. HSQC spectrum (300 MHz, acetone-d$_6$) of 1

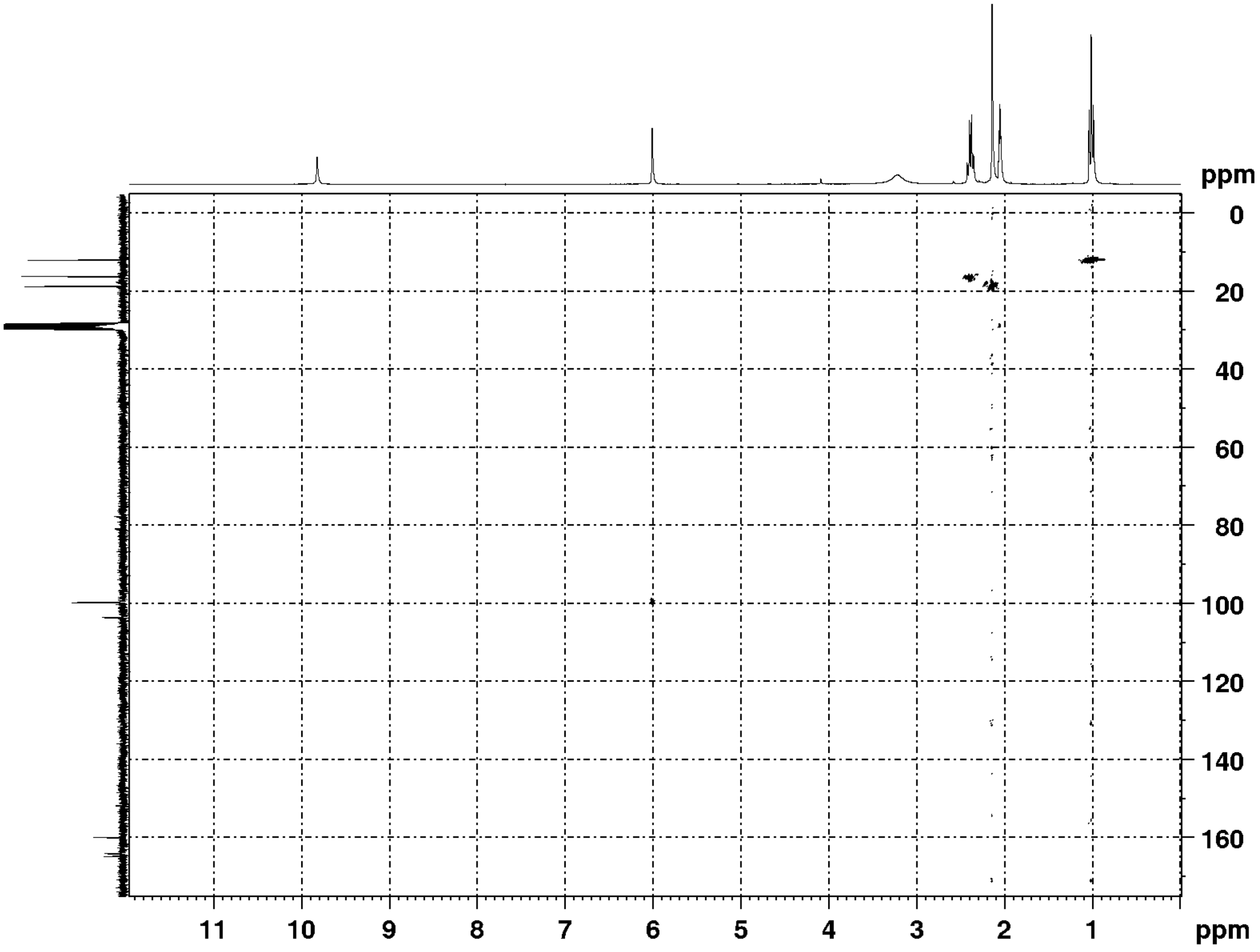


```
Current Data Parameters
NAME              TKPr-acetone-d6
EXPNO                        7001
PROCNO                          1

F2 - Acquisition Parameters
Date_                    20260430
Time                         9.44
INSTRUM                     spect
PROBHD     5 mm Dual 13C/1H Z3494/0355
PULPROG                    hsqcph
TD                           2048
SOLVENT                     CDCl3
NS                             16
DS                             16
SWH                      3591.954 Hz
FIDRES                   1.753884 Hz
AQ                      0.2850816 sec
RG                          645.1
DW                        139.200 usec
DE                           6.00 usec
TE                          297.2 K
CNST2                 145.0000000
d0                     0.00000300 sec
D1                     2.00000000 sec
d4                     0.00172414 sec
d11                    0.03000000 sec
DELTA                  0.00002100 sec
IN0                    0.00003680 sec

======== CHANNEL f1 ========
NUC1                           1H
P1                           7.50 usec
p2                          15.00 usec
PL1                         -4.00 dB
SFO1                  300.1318008 MHz

======== CHANNEL f2 ========
CPDPRG[2]                   garp4
NUC2                          13C
P3                           6.10 usec
p4                          12.20 usec
PCPD2                       65.00 usec
PL2                         -4.00 dB
PL12                        16.50 dB
SFO2                   75.4741638 MHz

F1 - Acquisition parameters
TD                             68
SFO1                     75.47416 MHz
FIDRES                 399.616364 Hz
SW                        180.021 ppm
FnMODE                       TPPI

F2 - Processing parameters
SI                           2048
SF                    300.1300076 MHz
WDW                         QSINE
SSB                             2
LB                              0 Hz
GB                              0
PC                           1.40

F1 - Processing parameters
SI                           2048
MC2                          TPPI
SF                     75.4677474 MHz
WDW                         QSINE
SSB                             0
LB                              0 Hz
GB                              0
```

Figure S9. HMBC spectrum (300 MHz, acetone-d$_6$) of 1

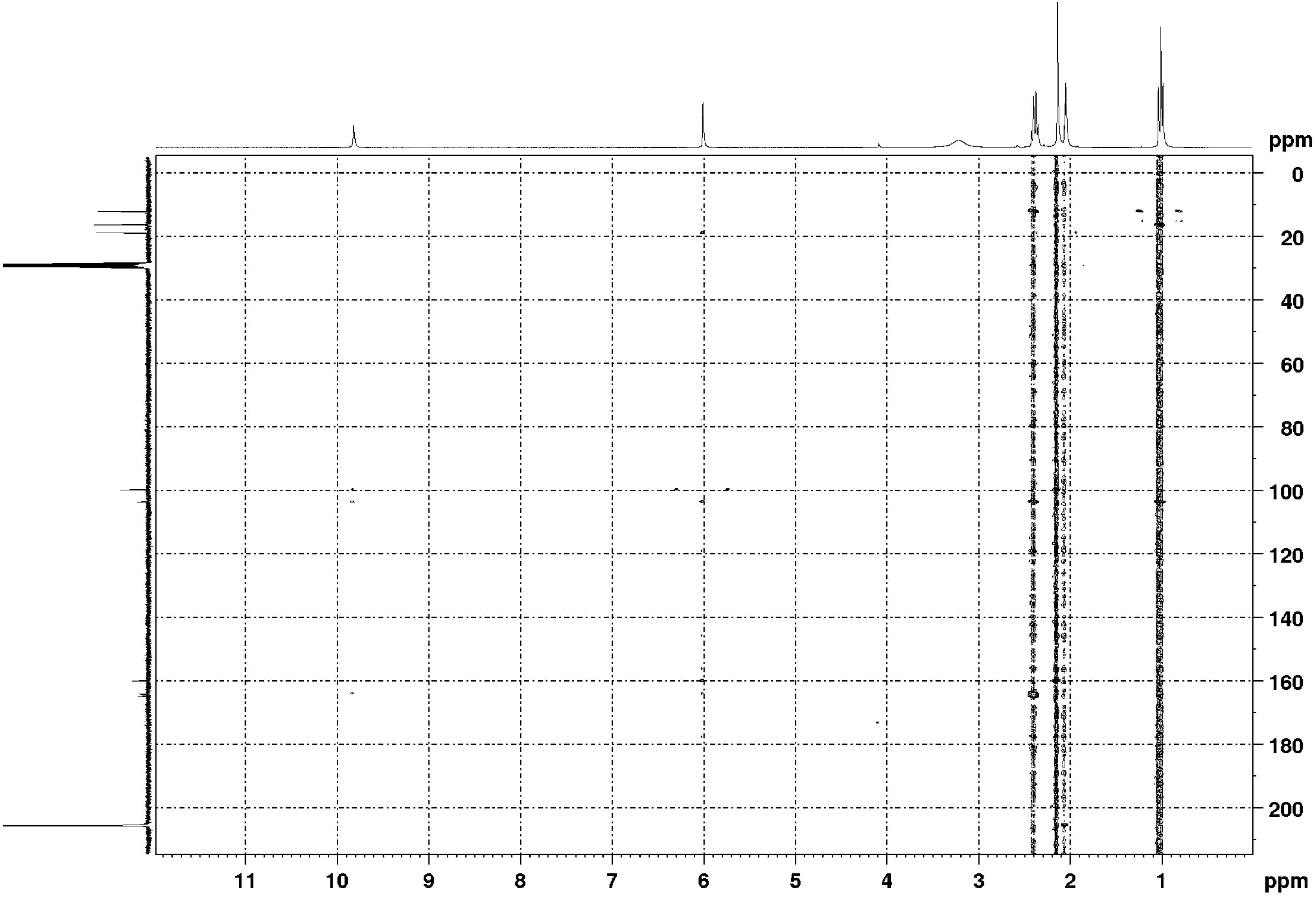

Figure S10. ^{1}H NMR spectrum (700 MHz, methanol -d_4) of 2

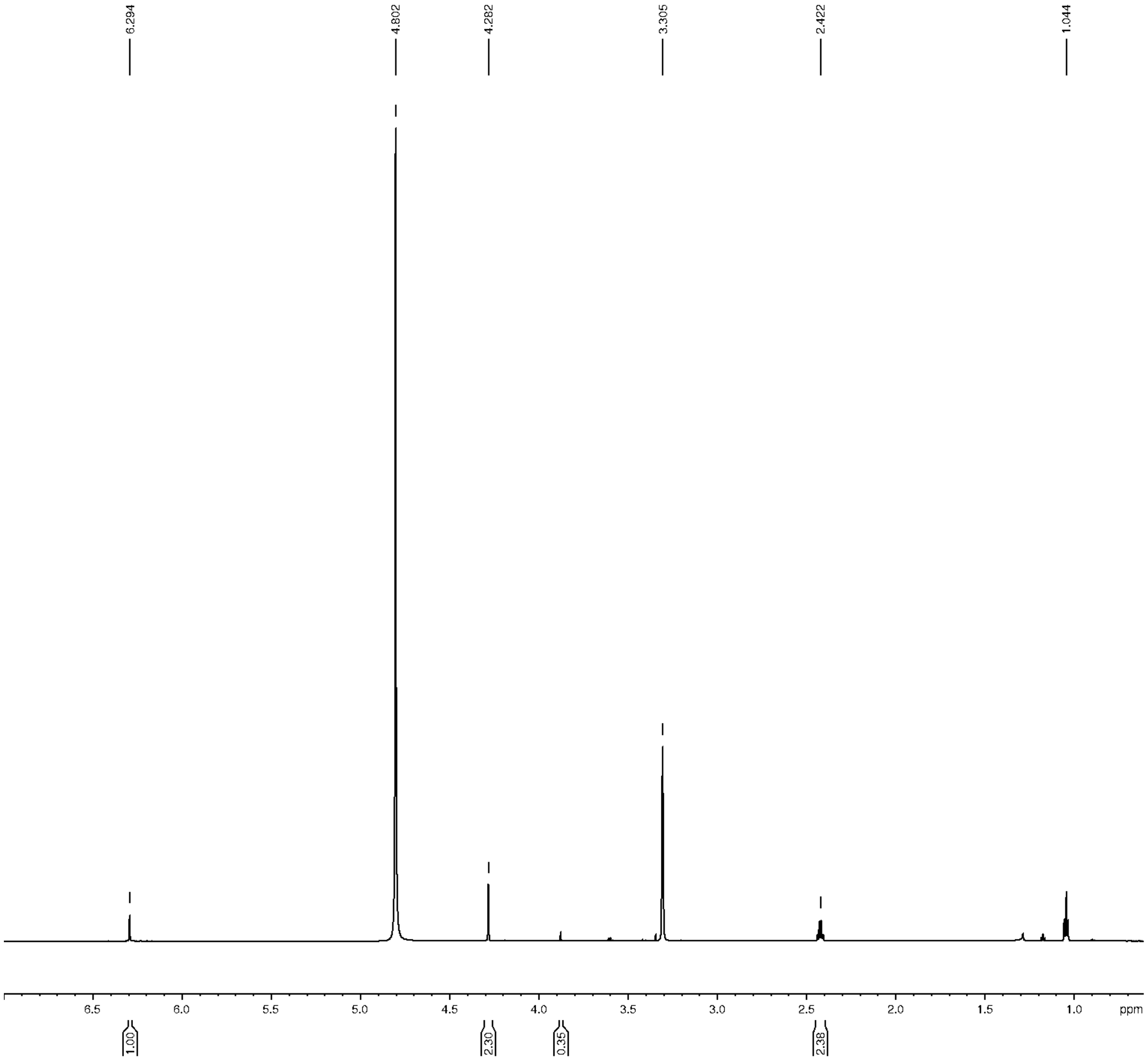


```
Current Data Parameters
NAME                TK-36h-2
EXPNO                     30
PROCNO                     1

F2 - Acquisition Parameters
Date_               20250405
Time                   17.25 h
INSTRUM                spect
PROBHD     Z134216_0006 (PA BBO 700S4 BB-H-D-05 :
PULPROG                 zg30
TD                     65536
SOLVENT                 MeOD
NS                        32
DS                         4
SWH                11160.714 Hz
FIDRES              0.340598 Hz
AQ                 2.9360127 sec
RG                     70.01
DW                    44.800 usec
DE                      6.50 usec
TE                     303.3 K
D1                0.10000000 sec
TD0                        1
SFO1             700.0054600 MHz
NUC1                      1H
P0                      5.33 usec
P1                     16.00 usec
PLW1             11.27900028 W

F2 - Processing parameters
SI                     65536
SF               700.0000165 MHz
WDW                       EM
SSB                        0
LB                      1.00 Hz
GB                         0
PC                      1.00
```

Figure S11. ^{13}**C NMR spectrum (700 MHz, methanol-d$_4$) of 2**

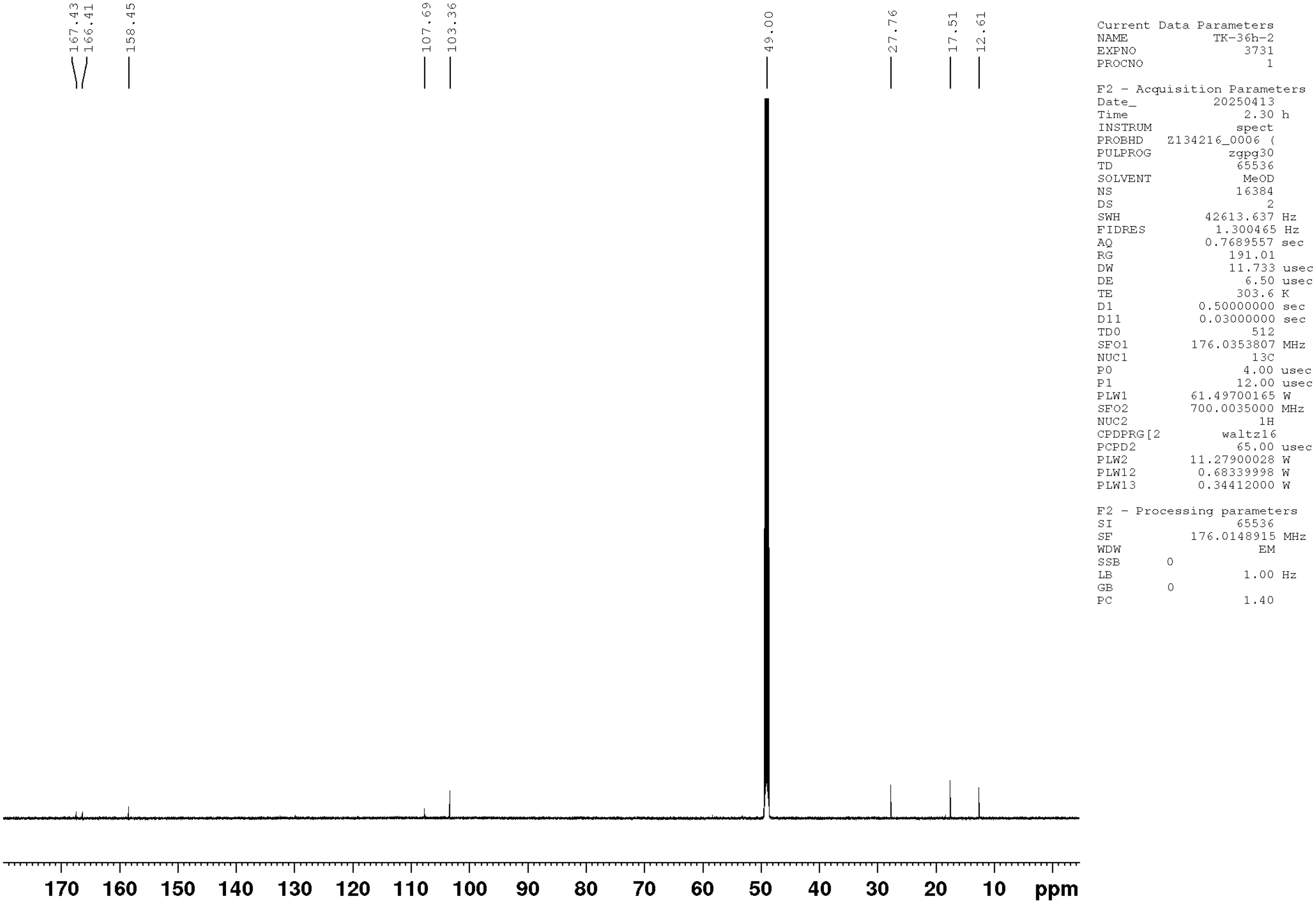

Figure S12. DEPT-135 spectrum (700 MHz, methanol-d$_4$) of 2

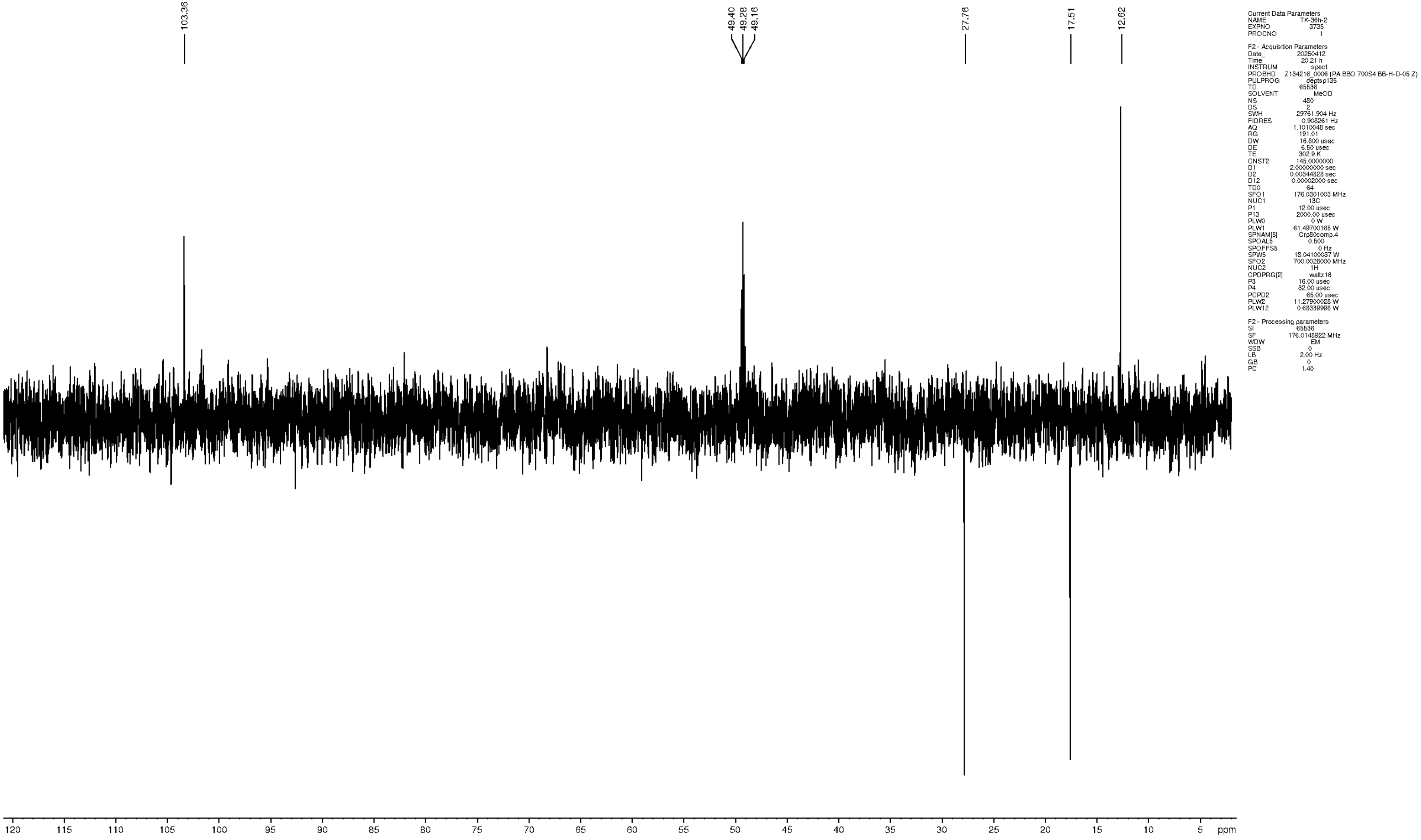

Figure S13. HMBC spectrum (700 MHz, methanol -d_4) of 2

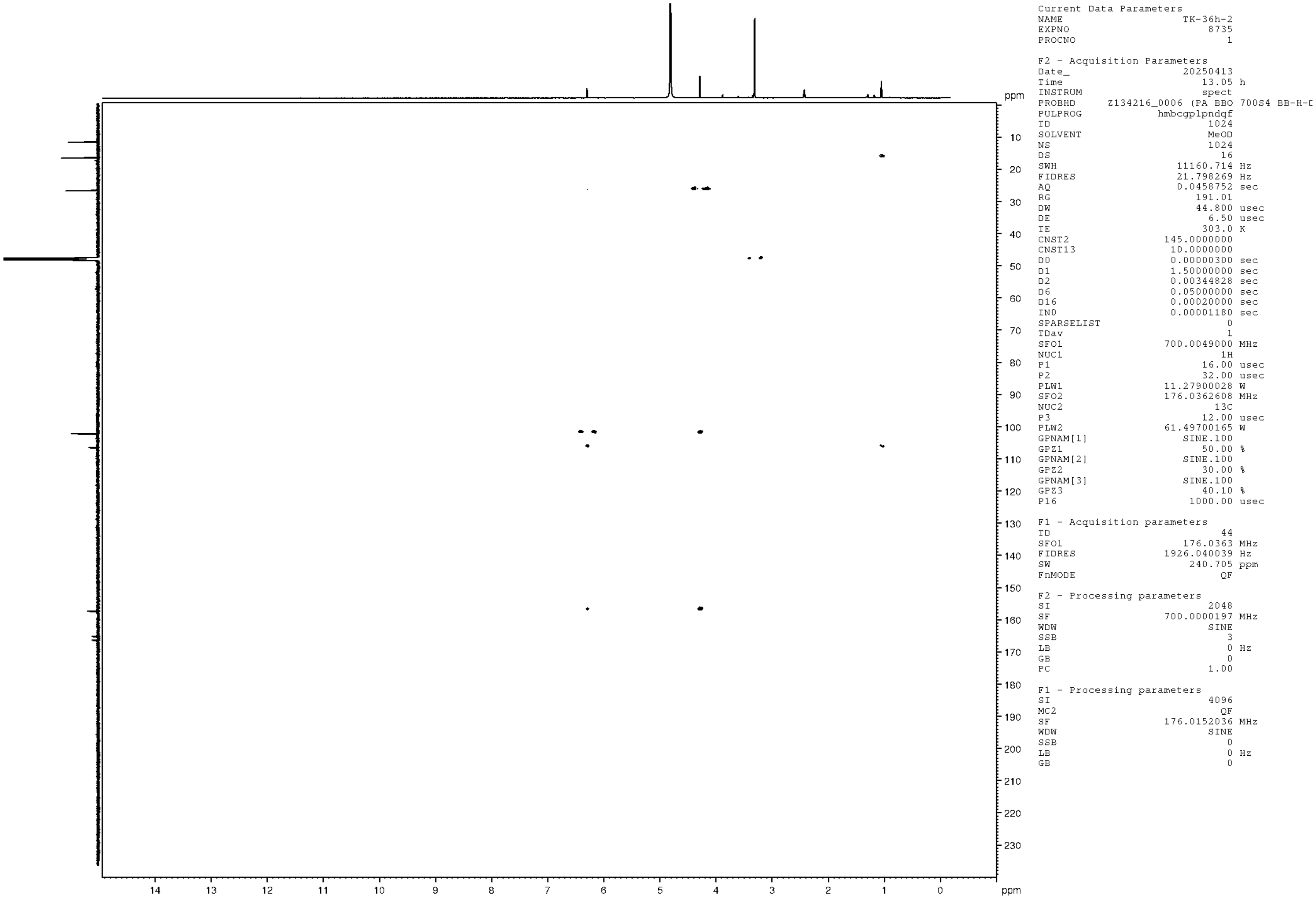

Figure S14. HSQC spectrum (700 MHz, methanol-d4) of 2

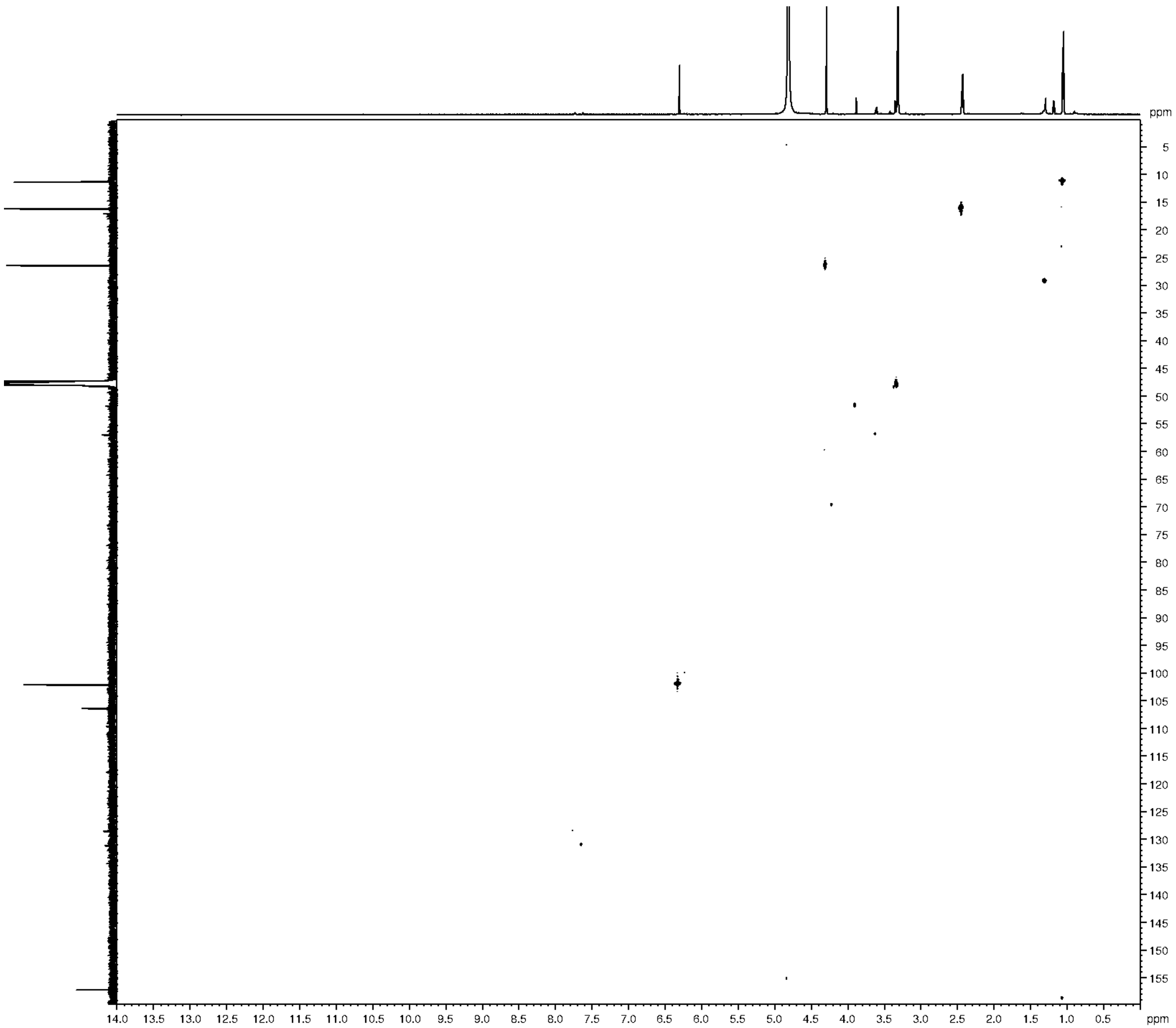

Figure S15. HR (–)ESI MS spectrum of 1

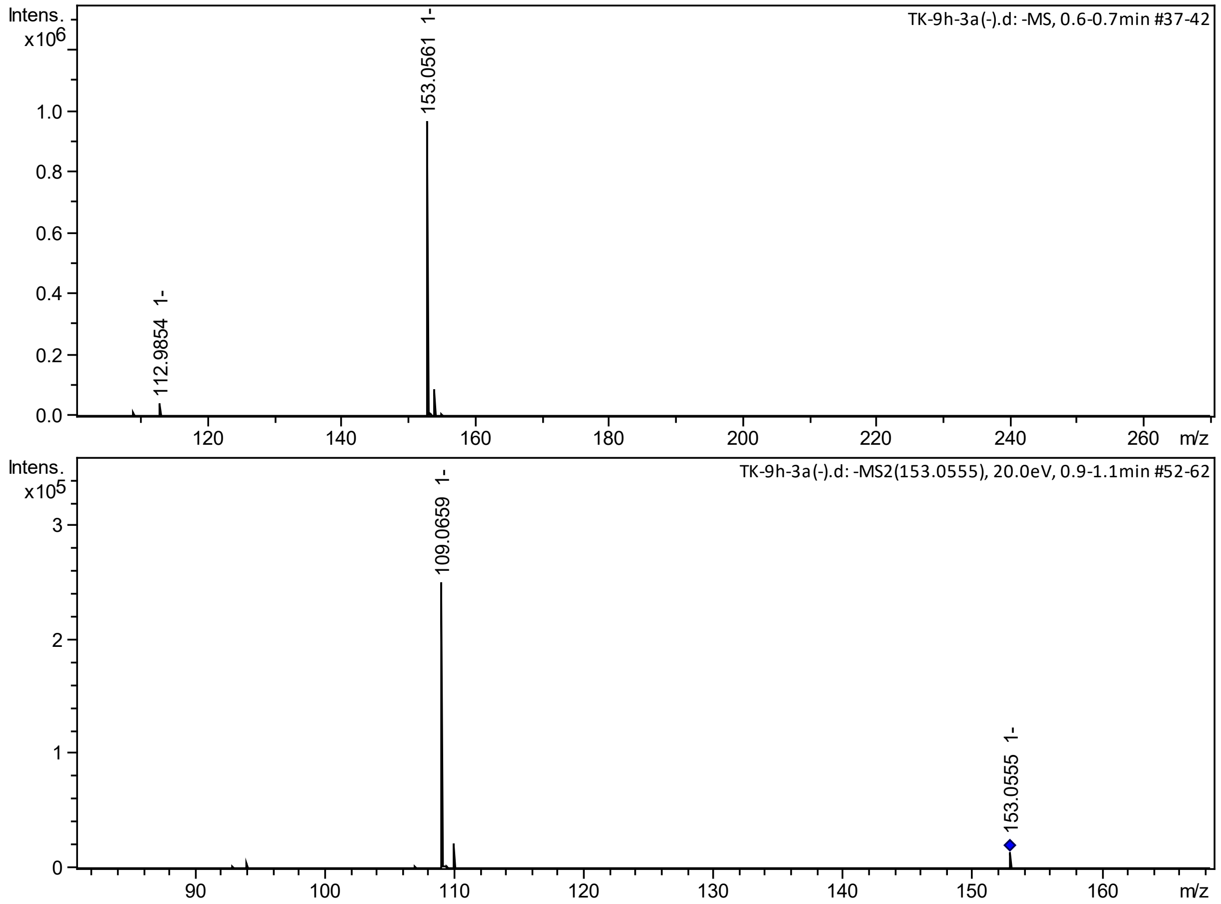

Figure S16. HR (–)ESI MS spectrum of 2

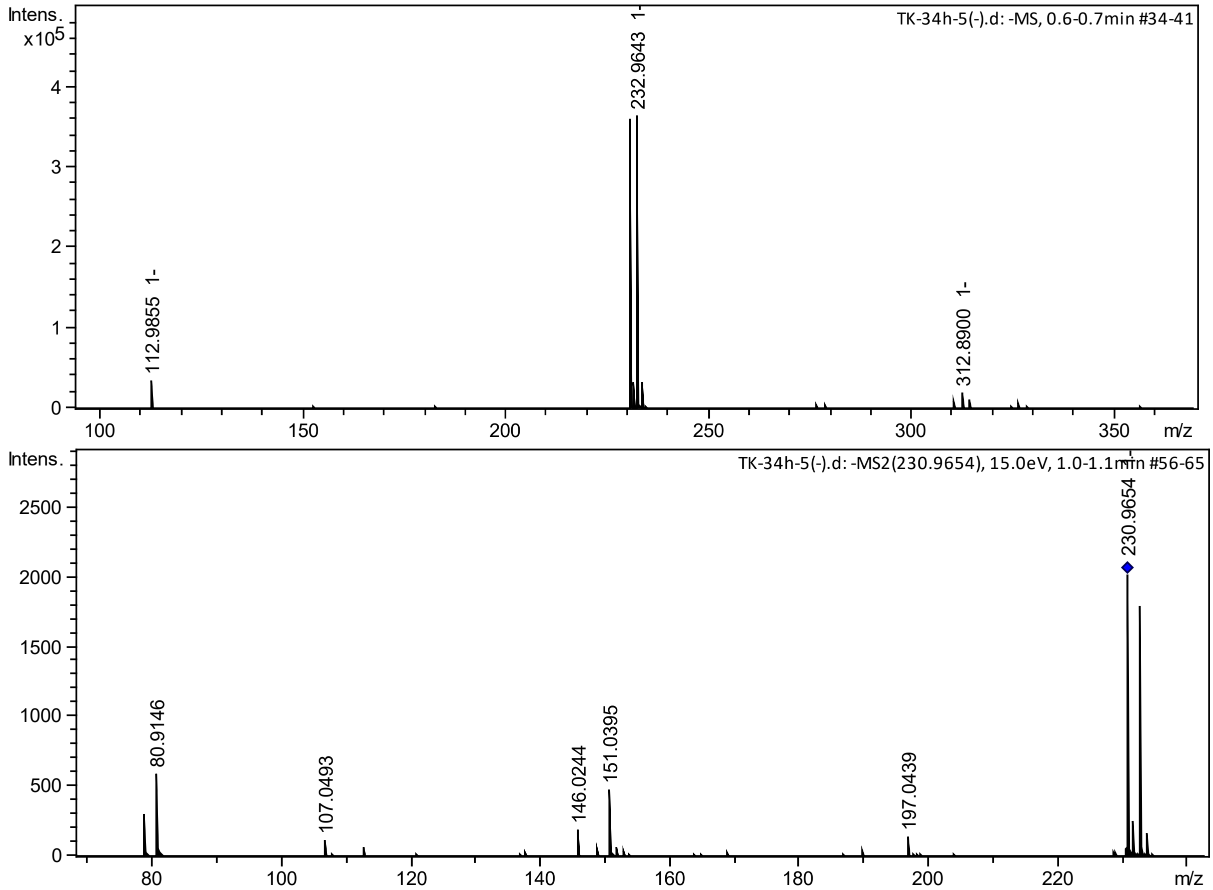

Figure S17. HR (–)ESI MS spectrum of mixture of unidentified products of bromination reaction

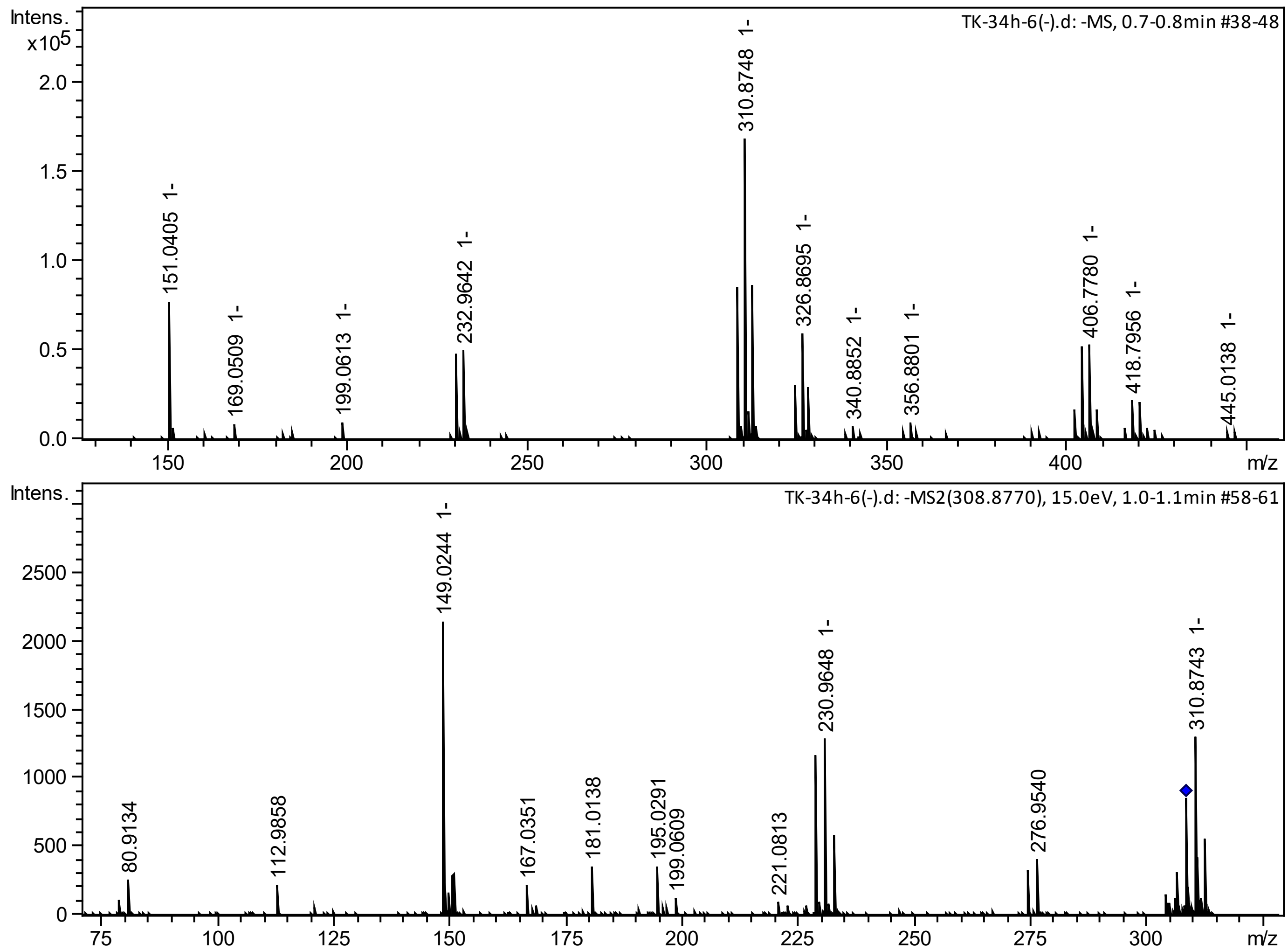

Table S4. ADMET properties of EHMP (1) and Br-EHMP (2)

Comp	Properties						
	CYP1A2	CYP2C19	CYP2C9	CYP2D6	CYP3A4	BBBP	
**1**	Non	Non	Non	Non	Non	Permeable	
**2**	Inhibitor	Non	Non	Non	Non	Permeable	
	Interactions with nuclear receptors and some others						
	NR-Aromatase	NR-ER	NR-AR	NR-AhR	NR-PPAR-gamma	SR-p53	SR-ARE
**1**	Non	Non	Non	Non	Non	Non	Non
**2**	Non	Non	Non	Non	Non	Non	Non
	In vivo toxicity						
	Reproductive toxicity	Mouse oral LD50 mg/kg	Mouse skin LD50 mg/kg	Rat oral LD50 mg/kg	Rat skin LD50 mg/kg	Cardio-toxicity	Carcino-genicity
**1**	Toxic	730.0	1388.0	1068.0	1534.0	Nontoxic	Nontoxic
**2**	Toxic	842.0	1226.0	466.0	1364.0	Nontoxic	Nontoxic
	Some calculated parameters and toxicities						
	QED	LogP	PAINS	Selective Target-Organ Or System Toxicity Single Exposure 58479-2019			Acute Toxicity Swallowed 56957-2016
**1**	0.661	1.22	not included	Hazard class 3 or Non-toxic: LD50 > 2000 mg/kg (rabbit skin)			Hazard class 4
**2**	0.792	1.8	not included	Hazard class 3 or Non-toxic: LD50 > 2000 mg/kg (rabbit skin)			Hazard class 4
